# Molecular mechanism of vimentin nuclear localization associated with the migration and invasion of daughter cells derived from polyploid giant cancer cells

**DOI:** 10.1186/s12967-023-04585-7

**Published:** 2023-10-13

**Authors:** Linlin Fan, Minying Zheng, Xinyue Zhou, Yongjun Yu, Yidi Ning, Wenzheng Fu, Jing Xu, Shiwu Zhang

**Affiliations:** 1 Department of Pathology, Tianjin Union Medical Center, Nankai University, Tianjin, 300071 People’s Republic of China; 2 Graduate School, Tianjin Medical University, Tianjin, 301617 China; 3 Department of Colorectal Surgery, Tianjin Union Medical Center, Nankai University, Tianjin, China; 4 Nankai University School of Medicine, Nankai University, Tianjin, 300071 China; 5 Department of General Surgery, Tianjin Union Medical Center, Nankai University, Tianjin, China

**Keywords:** Vimentin, Polyploid giant cancer cells, Colorectal cancer, SUMOylation, CDC42

## Abstract

**Background:**

Polyploid giant cancer cells (PGCCs), a specific type of cancer stem cells (CSCs), can be induced by hypoxic microenvironments, chemical reagents, radiotherapy, and Chinese herbal medicine. Moreover, PGCCs can produce daughter cells that undergo epithelial–mesenchymal transition, which leads to cancer recurrence and disseminated metastasis. Vimentin, a mesenchymal cell marker, is highly expressed in PGCCs and their daughter cells (PDCs) and drives migratory persistence. This study explored the molecular mechanisms by which vimentin synergistically regulates PGCCs to generate daughter cells with enhanced invasive and metastatic properties.

**Methods:**

Arsenic trioxide (ATO) was used to induce the formation of PGCCs in Hct116 and LoVo cells. Immunocytochemical and immunohistochemical assays were performed to determine the subcellular localization of vimentin. Cell function assays were performed to compare the invasive metastatic abilities of the PDCs and control cells. The molecular mechanisms underlying vimentin expression and nuclear translocation were investigated by real-time polymerase chain reaction, western blotting, cell function assays, cell transfection, co-immunoprecipitation, and chromatin immunoprecipitation, followed by sequencing. Finally, animal xenograft experiments and clinical colorectal cancer samples were used to study vimentin expression in tumor tissues.

**Results:**

Daughter cells derived from PGCCs showed strong proliferative, migratory, and invasive abilities, in which vimentin was highly expressed and located in both the cytoplasm and nucleus. Vimentin undergoes small ubiquitin-like modification (SUMOylation) by interacting with SUMO1 and SUMO2/3, which are associated with nuclear translocation. P62 regulates nuclear translocation of vimentin by controlling SUMO1 and SUMO2/3 expression. In the nucleus, vimentin acts as a transcription factor that regulates *CDC42*, cathepsin B, and cathepsin D to promote PDC invasion and migration. Furthermore, animal experiments and human colorectal cancer specimens have confirmed the nuclear translocation of vimentin.

**Conclusion:**

P62-dependent SUMOylation of vimentin plays an important role in PDC migration and invasion. Vimentin nuclear translocation and overexpressed P62 of cancer cells may be used to predict patient prognosis, and targeting vimentin nuclear translocation may be a promising therapeutic strategy for metastatic cancers.

**Supplementary Information:**

The online version contains supplementary material available at 10.1186/s12967-023-04585-7.

## Background

Although most patients with advanced colorectal cancer (CRC) undergo a combination of surgery, radiotherapy, and chemotherapy, a significant number die from disseminated disease several years after successful treatment of the primary tumor [1]. Thus, advanced CRC recurrence may result from the reactivation of rare and elusive CSCs hidden in specific ecological niches by specific signals [2]. CSCs are a subpopulation of cancer cells with self-renewal and tumor-initiating properties [3]. Polyploid giant cancer cells (PGCCs) have been described as CSCs in a variety of tumors, including breast [4], head and neck [5], and non-small cell lung cancers [6]. PGCCs temporarily stop dividing and maintain a low energy demand and response to external stimuli; therefore, they resist drug treatment. Previous studies have shown that autophagy, cell fusion, cell cycle arrest, endoreplication, and catastrophic cytokinesis are among the mechanisms underlying PGCCs formation [4, 7–9].

Treatment-induced PGCCs undergo protracted periods of proliferative arrest. Under certain favorable conditions or after the withdrawal of external factors, PGCCs rapidly activate and generate daughter cells via asymmetric division. Daughter cells are a distinct subpopulation of cancer cells that contribute to cancer heterogeneity [4]. Unlike conventionally sized diploid cancer cells, PGCC daughter cells exhibit mesenchymal properties with a high expression of mesenchymal cell markers, including N-cadherin, twist, slug, snail, and vimentin [10–12]. Vimentin is expressed in the nuclei of PGCCs and daughter cells (PDCs) and regulates gene expression and genomic stability by interacting directly with DNA through its N-terminal nonhelical structural domain [13, 14]. Vimentin expression is associated with aggressive and metastatic cancers. Moreover, PGCCs rely heavily on the vimentin network to maintain their expanded morphology and guide their continued migration [15]. A previous study has reported that vimentin influences nuclear alignment and is essential for generating daughter cells for outgrowth. This contributes to the development of therapeutic resistance and, ultimately, disease recurrence and metastasis [16]. However, little is known about the exact mechanism by which vimentin is associated with the invasion and migration of daughter cells generated by PGCCs.

Small ubiquitin-like modification (SUMOylation) is a post-translational modification that reversibly and dynamically targets lysine residues of a wide range of proteins. SUMOylation is involved in various functional cellular events, including mitotic chromosome segregation, protein translocation, and degradation [17–19]. Previous studies have reported that small ubiquitin-like modifier protein (SUMO) is highly expressed in PDCs [20]. Recent findings suggested that the protein inhibitor of activated STAT-1 (PIAS1), a SUMO E3 enzyme, specifically links SUMO to the K439 and K445 residues of vimentin [21]. Unlike the UBC9 substrate, which is normally SUMOylated on its consensus motif, the receptor lysine residues found on vimentin are highly conserved but are located in nonconsensus sequence motifs [21]. Interestingly, SUMOylation of vimentin favors cell proliferation and migration, which may increase the proliferation and invasiveness of cancer cells.

Daughter cells derived from PGCCs have strong proliferative, migratory, and invasive abilities, which play important roles in the metastasis and recurrence of malignant tumors. In the last decade, nanomaterials from various natural species (leaves of *Olea europaea* trees and *Scutellaria baicalensis*) have been applied in the diagnosis and therapy of cancer [22–25]. In addition, multivesicular vesicle nanocarriers contain at least two functionalities that can attack two or three targets in tumor cells and reduce drug resistance and disease recurrence [26, 27]. Such multivesicular vesicle nanocarriers may precisely deliver drugs to target PGCCs and their daughter cells and inhibit the metastasis and recurrence of PDCs by regulating the subcellular location of vimentin.

In the present study, we investigated the mechanisms underlying high vimentin expression and SUMOylation in PGCCs to form daughter cells with greater invasive and metastatic capacities. P62-dependent SUMOylation of vimentin results in its nuclear translocation and plays an important role in PDCs migration and invasion. Furthermore, we validated our findings using highly relevant CRC clinical samples and investigated the clinical data to determine the relationship between the number of PGCCs, percentage of vimentin nuclear positivity, and degree of tumor differentiation.

## Methods

### Culture of colon cancer cell lines

Hct116 and LoVo human colon cancer cell lines were obtained from the American Type Culture Collection (ATCC, Manassas, VA, USA). Hct116 and LoVo cells were cultured on the 1640 medium (Gibco, Thermo Feil Technology Co., Ltd., Suzhou, China) supplemented with 10% fetal bovine serum (FBS; Gibco, Life Technologies, New Zealand) and 1% penicillin–streptomycin (Gibco, Life Technologies, USA) in an incubator at 37 °C under 5% CO_2_.

### Arsenic trioxide (ATO) induces PGCC formation

PGCCs can be induced by hypoxia, chemical reagents, radiotherapy, or Chinese herbal medicines. In the present study, we used ATO to induce the formation of PGCCs. Hct116 and LoVo cells were cultured until they reached approximately 80–90%. Then, 32 µM ATO (ATO was presented by Professor Gao Yu from the Department of Pharmacy, Beijing Institute of Radiological Medicine) was added in the medium with serum for 48 h for Hct116 cells and for 24 h for LoVo cells. After ATO treatment, most cancer cells were killed and only scattered PGCCs survived. Cells were rinsed with phosphate-buffered saline (PBS; Gibco, Thermo Fisher Technology, Suzhou, China) and cultured in a serum-containing medium. After 7–10 days, the PGCCs began to produce daughter cells via asymmetrical division. After three ATO treatments, PGCCs comprised 20–30% of the total cells, with 70–80% of the population being daughter cells derived from PGCCs.

### Immunocytochemical (ICC) staining

Hct116 and LoVo cells treated with or without ATO were cultured on slides in a six-well plate until they reached 80–90% confluence. The cells were treated with 4% pre-cooled paraformaldehyde for 30 min and then exposed to endogenous peroxidase inhibitors (Zhongshan, Beijing, China) and goat serum (Zhongshan) at room temperature for 15 and 30 min, respectively. Primary antibodies (Additional file 1: Table S1) were added to the cells and incubated overnight at 4 °C. The next day, the cells were incubated with appropriate secondary antibodies and horseradish peroxidase-labeled streptomyces ovalbumin working solution (Zhongshan Company) for 30 and 15 min, respectively. 3,3′-Diaminobenzidine (DAB) was used for color development (Zhongshan, Beijing, China). Nuclei were stained with hematoxylin, and the tablet was sealed after dehydration with an alcohol gradient.

### Wound healing

Cell migration was evaluated using wound healing experiments. Briefly, Hct116 and LoVo cells treated with or without ATO were incubated in a six-well plate, and the surfaces of the plates were scratched using sterile pipette tips to create wounds. PBS was used to remove the suspended cells, and the specimens were cultured in serum-free medium at 37 °C under 5% CO_2_. Photographs were taken at 0 and 24 h, and the scratched areas were measured using the ImageJ software.

### Transwell migration and invasion assay

A transwell cell migration assay (8 μm; Corning Company, 24-well plate) was performed. Cells (5 × 10^4^) were inoculated with 200 µL medium supplemented with 1% FBS in the upper compartment and 600 µL medium supplemented with 20% FBS in the lower compartment. After incubation for 24 h, the cells were fixed with anhydrous methanol for 30 min and stained with 0.1% crystal violet for 30 min. A transwell invasion assay was performed to evaluate the invasion ability of PDCs. Briefly, cell suspensions were generated with 5 × 10^5^ cells per well and then inoculated into the wells in 200 µL medium without FBS. The insert was pre-coated with the BD Matrigel Basement Membrane Matrix (Corning). Growth medium with 20% FBS was used as a chemical inducer and added to the bottom chamber, and the plates were incubated under 5% CO_2_ at 37 °C for 12 h. After removing the FBS-free medium and the upper chamber, the Transwell inserts and invasive cells were fixed in methanol for 30 min and stained with 0.1% crystal violet for 30 min. The cells that penetrated the membrane were counted in five visual fields.

### Plate-based colony formation assay

A plate clone formation assay was performed to evaluate the proliferative ability of PDCs. Briefly, the cells were seeded at densities of 30, 60, and 120 cells/well in 12-well plates. Cells were cultured at 37 °C under 5% CO_2_ for 2 weeks. When visible cell colonies appeared at the bottom of the plate, the samples were fixed with anhydrous methanol for 30 min and stained with 0.1% crystal violet for 30 min. The number of cell colonies was counted using a microscope (a cluster containing ≥ 50 cells was counted as a single colony). The colony formation efficiency was defined as the number of inoculated cells.

### Western blot (WB) analysis

When the cells reached 80–90% confluence, Hct116 cells, LoVo controls, and PDCs were collected. The cells were lysed on ice with approximately 100 µL of lysis solution (Roche, Germany) for 30 min and then centrifuged at 4 °C and 14,000 rpm for 30 min. After determining the protein concentrations in the samples, the proteins were boiled at 100 °C for 10 min and separated on a 10% sodium dodecyl sulfate polyacrylamide gel. Protein bands were transferred onto polyvinylidene difluoride membranes (GE Healthcare, USA), which were blocked with 5% skim milk (BD Biosciences, USA) at room temperature for 90 min. The membranes were then incubated overnight with the respective primary antibodies (Additional file 1: Table S1) at 4 °C. Subsequently, the membranes were incubated with secondary antibodies at room temperature for approximately 2 h. β-Actin and histone-H3 were used as protein loading controls. Protein expression was detected using a ChemiDoc Imaging System (Bio-Rad, USA).

### Cell transfection

Cells were inoculated into six-well plates. When the cells reached 30–50% confluence, siRNAs were transfected into the cells using Lipofectamine 2000 transfection reagent (Life Technologies, Shanghai, China) according to the manufacturer’s instructions. Transfection reagents were diluted with Opti-MEM I (Gibco) in a serum-free medium. Cell samples were collected 72 h after transfection to detect the inhibition efficiency of the targeted proteins using WB analysis. The siRNA sequences used are listed in Additional file 1: Tables S2–S7.

### Co-immunoprecipitation

The cells were collected in a new cold Eppendorf tube and lysed with immunoprecipitation lysis buffer (87787, Thermo Fisher Scientific, Guangzhou, China) for 30 min. Agar glycoprotein beads A/G homogenate (20421, Thermo Fisher Scientific) were added with 500 µL lysate in each tube and rinsed three times. The lysed samples were added to the beads and incubated for 30 min. 10% of the total volume of the supernatant was used as the input and the remaining sample volumes were divided into two parts. One part was supplemented with normal mouse immunoglobulin (Ig) G (A7028, Biyuntian), and the other with a vimentin antibody against the target protein. After mixing, the samples were slowly shaken overnight at 4 °C for incubation. The samples were added to the washed agar-agar beads and incubated for 2 h at 4 °C. After washing and centrifugation, the immunoprecipitation results were confirmed by WB blotting using an anti-vimentin antibody. The experiments were independently repeated at least three times.

### Ginkgolic acid (GA) and MG132 treatment

ATO-treated cells were seeded in six-well plates until they reached 80% confluence. MG132 (carbobenzoxyl-l-leucyl-l-leucyl-l-leucine) (10 µM, Selleck Chemicals) was added, and the cells were incubated for 6 h and then collected for further experiments. Approximately 20 µM of ginkgolic acid (GA) (15:1, MedChem Express, USA) was added to control cells and PDCs for 24 h, and the samples were evaluated using WB analysis and other assays described herein.

### Chromatin immunoprecipitation (ChIP) assay and data analysis

A ChIP assay was performed to identify the genes interacting with vimentin in the PDCs, and the cells were manipulated using a Pierce Magnetic ChIP kit 26157 (Thermo Fisher Technologies). Cells were treated with 1% formaldehyde to crosslink transcription factors to chromatin DNA and suspended in lysis buffer supplemented with a protease inhibitor mixture. The cells were then ultrasonicated to cleave chromatin DNA into 200 and 1000 base pairs for sequencing. For ChIP DNA data analysis, sequencing, and library preparation were performed by Novogene Technology Co. Ltd. (Tianjin, China). The location of peaks around transcription start sites can predict protein-gene interaction sites. Genes associated with different peaks were identified, and gene ontology (GO) and Kyoto Encyclopedia of Genes and Genomes (KEGG) enrichment analyses were performed. To evaluate the interaction between vimentin and its downstream genes, a spliced DNA fragment was extracted by reverse cross-linking using specific gene primers for further amplification by polymerase chain reaction (PCR).

### Real-time PCR (qPCR) analysis

Total RNA was extracted from cells using TRIzol reagent and treated with DNase I (Thermo Fisher Scientific). qPCR was performed using the LightCycler480 instrument (Roche, Germany) to amplify downstream gene-specific sequences. The PCR conditions were set according to the instructions provided in the SYBR Green Kit (Roche). Primers used in this study were synthesized by Genewiz (Suzhou, China). Primer sequences were selected based on Homo sapiens mRNA (GenBank accession number: AF498962). The qPCR primer sequences are provided in Additional file 1: Table S8.

### Immunofluorescent staining

Cells in 6-well plates were fixed with 4% formaldehyde at room temperature for 15 min. After washing with PBS, the cells were permeabilized with 0.25% Triton-X at room temperature for 15 min and incubated with 5% bovine serum albumin blocking solution for 1 h. The cells were then incubated overnight with antibodies against SUMO1 and SUMO2/3. A fluorescent secondary antibody conjugated to Alexa Fluor 594™ was then added, and the DNA was stained using the Hoechst 33342 reagent. Images were captured using a fluorescence microscope.

### Animal experiment

Twenty male BALB/c nude mice (aged 4–5 weeks, Beijing Weitong Lihua Co., Ltd.) were divided into four groups (five mice per group). Mice in each group were injected with a 200 µL tumor cell (1 × 10^6^ cells) suspension containing one of the following: (i) Hct116 control cells, (ii) Hct116 PDCs, (iii) LoVo control cells, and (iv) LoVo PDCs. The tumor size was measured daily after the tumor diameter reached 0.25 cm. The animals were euthanized 28 days after inoculation. Paraffin-embedded tumor tissues were used for hematoxylin and eosin (H&E) and immunohistochemical (IHC) staining, and fresh tumor tissues were used for WB analysis. The animal experiments were approved and supported by the Institutional Animal Care and Use Committee of Tianjin Union Medical Center (approval number: 2022B37).

### H&E staining

The tumor tissues were fixed in formalin for 48 h at room temperature and embedded in paraffin. Tissues were sectioned at a thickness of 4 μm, baked at 71 °C for 2 h, de-paraffined in xylene for 30 min, and rehydrated with a descending ethanol concentration series. Sections were stained at room temperature with 0.2% hematoxylin (Guangzhou Basso, China) for 3 min and 0.5% eosin for 2 min. After staining, sections were dehydrated and mounted on coverslips.

### IHC staining

Xylene was used to deparaffinize the slides, which were rehydrated using a gradient alcohol solution. To restore the antigen, the slices were immersed in a boiling citrate buffer solution (Zhongshan Company) for 5 min. Next, endogenous peroxidase inhibitors were added to block endogenous peroxidase activity, along with goat serum to block nonspecific background staining. Sections were incubated overnight with the respective primary antibodies (Additional file 1: Table S1) at 4 °C and subjected to a staining procedure similar to ICC staining. Staining was considered positive when the cytoplasm and nucleus appeared brownish-yellow.

### Human CRC samples

In total, 155 paraffin-embedded CRC tissue samples were collected from the Pathology Department of Tianjin Union Medical Center. Based on the pathological results, patients were divided into three groups: Group I, 55 patients with well-differentiated CRC cells; Group II, 50 patients with moderately differentiated CRC cells; and Group III, 50 patients with poorly differentiated CRC cells. This study was approved by the Tianjin Union Medical Center Hospital Review Committee, and patient information was kept confidential.

### Statistical analysis

Statistical software (SPSS 16.0; IBM Corporation, New York, USA) was used to analyze all data in this study. All histogram data are expressed as means ± standard deviations. Statistical significance was set at *P* < 0.05.

## Results

### Daughter cells derived from PGCCs showed strong proliferation, migration, and invasion abilities

**Fig. 1:**
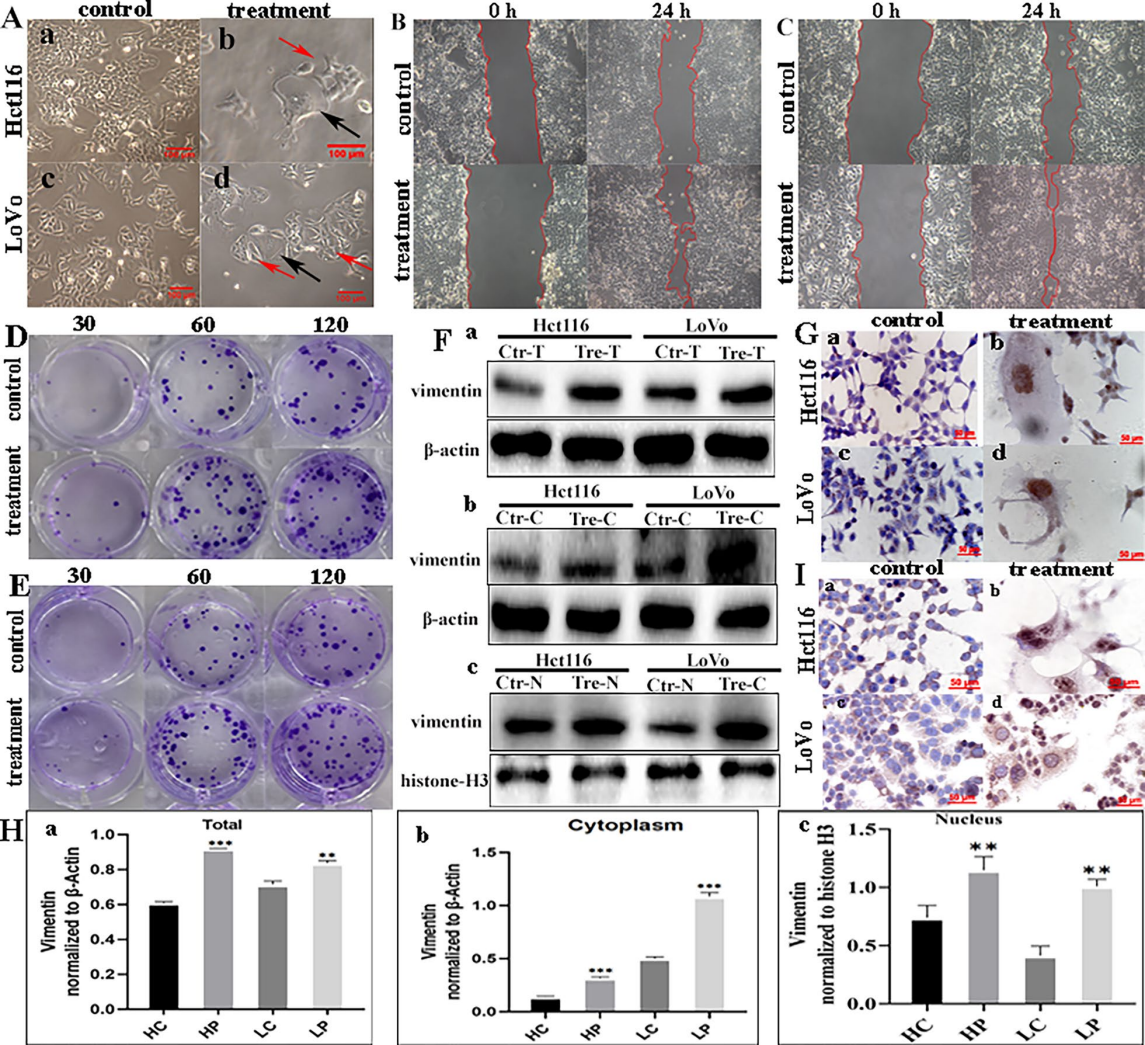
Morphologic characteristics and functional analysis of PGCCs.** A** PGCC induction and daughter cell generation following treatment (×100). **a** Hct116 control cells; **b** Hct116 PGCC (black arrow) and daughter cells (red arrow) after recovery from treatment; **c** LoVo control cells; **d** LoVo PGCC (black arrow) and daughter cells (red arrow) after recovery from treatment. **B** Wound healing assay in Hct116 cells before and after treatment at 0 and 24 h (×100). **C** Wound healing assay in LoVo cells before and after treatment at 0 and 24 h (×100). **D** Colony formation assays of 30, 60, and 120 Hct116 cells before and after treatment. **E** Colony formation assays of 30, 60, and 120 LoVo cells before and after treatment. **F** Western blot analysis of vimentin. **a** Total protein levels of vimentin, **b** cytoplasmic levels of vimentin, and **c** nuclear levels of vimentin. **G** ICC staining was performed to evaluate vimentin expression in Hct116 and LoVo cells before and after treatment (×200). **H** ICC staining to evaluate PIAS1 expression in Hct116 and LoVo cells before and after treatment (×200). **I** Grey value analysis of western blots. All data represent the means ± standard errors of the means of at least three independent experiments. **a** Total protein levels of vimentin, **b** cytoplasmic levels of vimentin, and **c** nuclear levels of vimentin. *P* values were calculated using a one-way analysis of variance. *P < 0.05; **P < 0.01; ***P < 0.001. *PGCCs* polyploid giant cancer cells, *PDCs* PGCCs with daughter cells, *HC* Hct116 control cells, *HP* Hct116 PDCs after treatment, *LC* LoVo control cells, *LP* LoVo PDCs after treatment, *ICC* immunocytochemical

When the tumor cells were exposed to 32 µM ATO (Fig. 1A, a and c), most were killed and only a few large cells survived. The nuclear morphology of the surviving cells as well as their increased size and elevated DNA content suggested that the cells developed into multinucleated PGCCs (Fig. 1A, b and d). With an increase in the recovery culture time, many smaller daughter cells proliferated rapidly via asymmetric division (Fig. 1A, b and d). The results of the wound healing assay confirmed the enhanced migratory capacity of these regenerating daughter cells compared to that of the untreated cells (Fig. 1B, C), and the difference between the control cells and PDCs was statistically significant (Additional file 4: Fig. S1A). The plate cloning assay was then used to detect the cell proliferative ability. The number of clones formed in 30, 60, and 120 Hct116 and LoVo control cells was less than that of the 30, 60, and 120 PDCs (Fig. 1D, E), and the differences were statistically significant (Additional file 4: Fig. S1B, a and b). Compared to control cells, PDCs showed increased migration, invasion, and proliferation.

### Vimentin expression and nuclear translocation is associated with PDC proliferation, migration, and invasion abilities

Vimentin is known to maintain cellular integrity, provide resistance to stress, and is an essential marker of the epithelial–mesenchymal transition. Therefore, the expression and subcellular localization of vimentin were assessed using IHC and WB in Hct116 (Fig. 1F, G) and LoVo cells (Fig. 1F, G). The subcellular localization of vimentin in these cells was concentrated in the nucleus of PGCCs (Fig. 1G, b and d), whereas there was no obvious vimentin expression in the control cells (Fig. 1G, a and c). The total (Fig. 1H, a), cytoplasmic (Fig. 1H, b), and nuclear (Fig. 1H, c) expression of vimentin in PDCs was significantly higher than that in control cells.

### SUMOylaytion promotes the nuclear localization of vimentin in PDCs

**Fig. 2:**
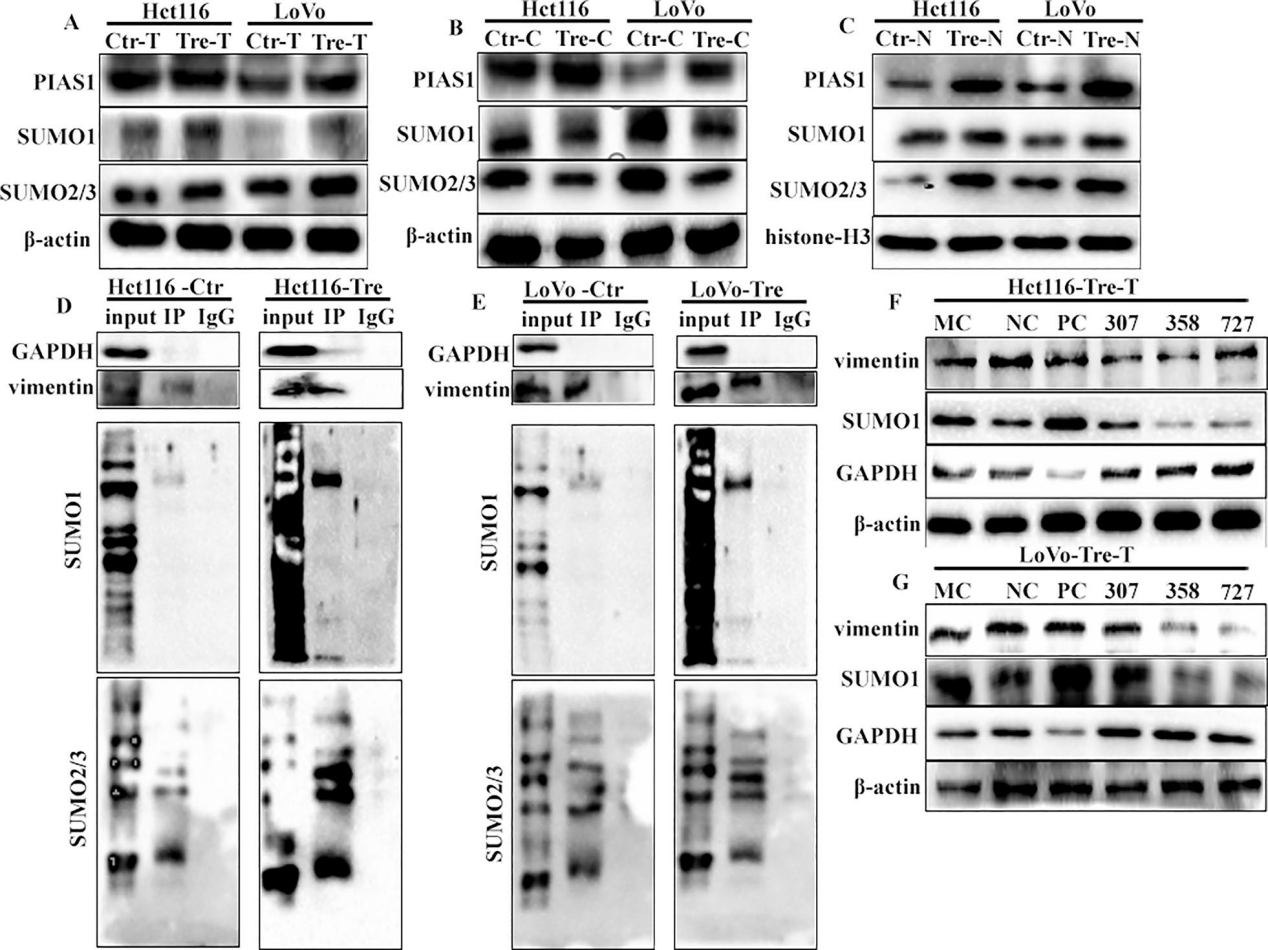
SUMOylation promotes the nuclear translocation of vimentin in PDCs.** A** Western blot analysis of SUMOylated vimentin-related total cell protein levels (PIAS1, SUMO1, and SUMO2/3). **B** Western blot analysis of SUMOylated vimentin-related cytoplasmic protein levels (PIAS1, SUMO1, and SUMO2/3). **C** Western blot analysis of SUMOylated vimentin-related nuclear protein levels (PIAS1, SUMO1, and SUMO2/3). **D** Results of vimentin coimmunoprecipitation in Hct116 control cells and Hct116 PDCs (anti-vimentin antibody was used for immunoprecipitation). **E** Results of vimentin co-immunoprecipitation in LoVo control cells and LoVo PDCs (an anti-vimentin antibody was used for immunoprecipitation). **F** Total expression of SUMO1 and vimentin in Hct116 PDCs after transfection with three siRNA-SUMO1 sequences (307, 358, and 727). **G** Total expression of SUMO1 and vimentin in LoVo PDCs after transfection with three siRNA-SUMO1 sequences (307, 358, and 727). *SUMO* small ubiquitin-like modification, *PDCs* polyploid giant cancer cells with daughter cells

SUMO1/2 and PIAS1 have been reported to promote SUMOylation of vimentin, which plays an important role in enhancing breast cancer metastasis [21]. Therefore, detecting the expression of SUMOylation-related proteins in PDCs can help clarify the molecular mechanisms underlying vimentin nuclear localization. The subcellular localization of PIAS1, a SUMO E3 enzyme, was detected using ICC staining. PIAS1 expression was observed only in the cytoplasm of control cells (Fig. 1I, a and c). However, in PDCs, vimentin expression was observed in the nuclear and perinuclear regions (Fig. 1I, b and d). The WB results showed that nuclear levels of PIAS1, SUMO1, and SUMO2/3 were significantly increased in PDCs compared to those in control cells (Fig. 2A–C) (*P* < 0.01, Additional file 4: Fig. S1C–E). Protein immunoprecipitation also confirmed that SUMO1 and SUMO2/3 interact with vimentin in PDCs (Fig. 2D, E).

### Inhibition of SUMOylation modifications prevents vimentin nuclear translocation

**Fig. 3:**
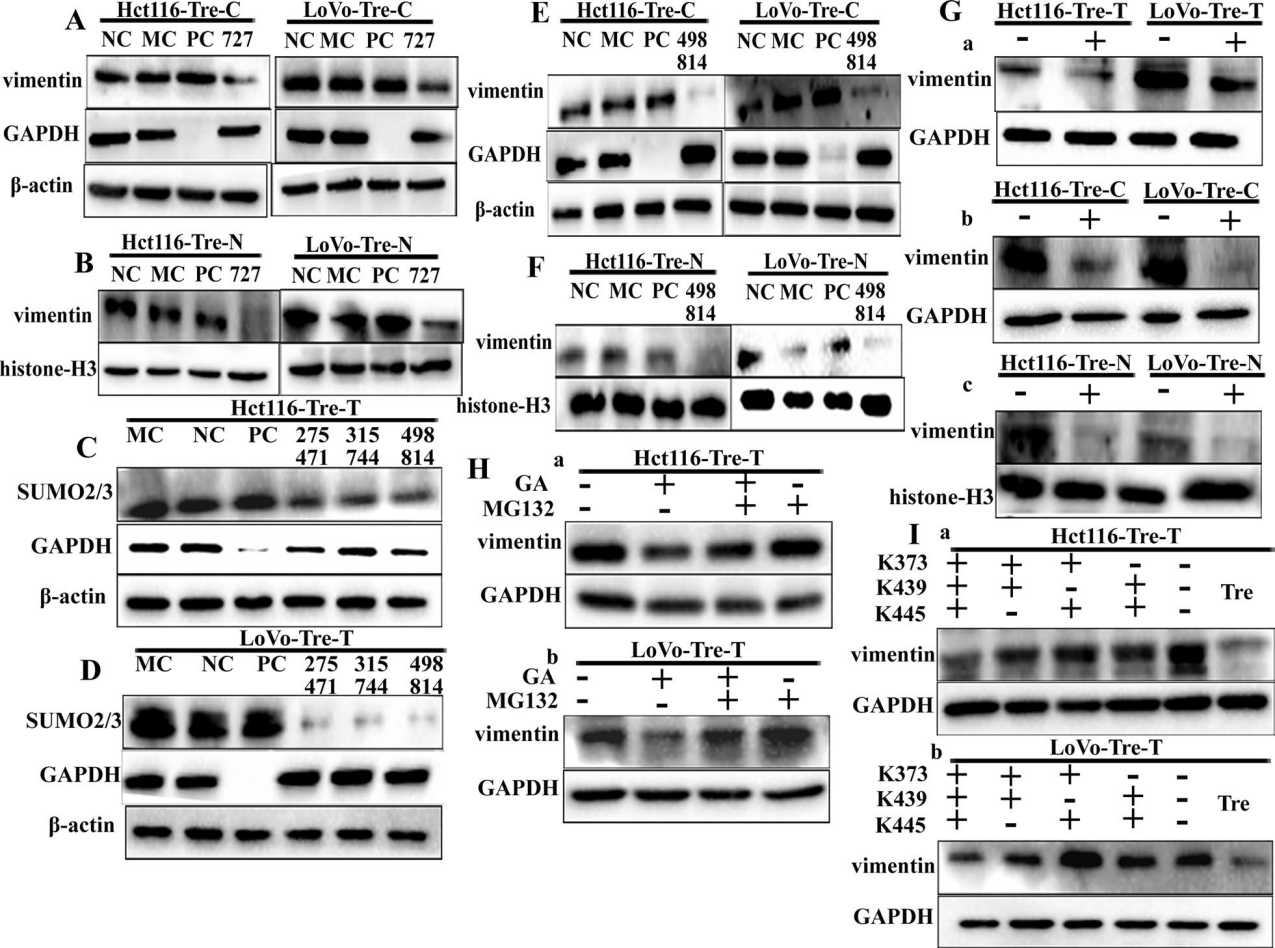
Expression of vimentin in PDCs after inhibition of SUMOylation and ubiquitination.** A** Cytoplasmic vimentin expression in Hct116 and LoVo PDCs after siRNA-SUMO1 transfection. **B** Nuclear vimentin expression levels in Hct116 and LoVo PDCs after siRNA-SUMO1 transfection. **C** Total SUMO2/3 expression levels in Hct116 PDCs after transfection with siRNA-SUMO2/3 (275 471, 315 744, 498 814) transfection. **D** Total vimentin expression levels in LoVo PDCs after siRNA-SUMO2/3 (275 471, 315 744, 498 814) transfection. **E** Cytoplasmic vimentin expression levels in Hct116 and LoVo PDCs after siRNA-SUMO1 transfection. **F** Nuclear vimentin expression levels in Hct116 and LoVo PDCs after siRNA-SUMO1 transfection. **G** (**a**) Total vimentin expression levels in Hct116 PDCs and LoVo PDCs after GA. **b** Cytoplasmic vimentin expression levels in Hct116 PDCs and LoVo PDCs after GA. **c** Nuclear vimentin expression levels in Hct116 PDCs and LoVo PDCs after GA. **H** (**a**) Vimentin expression levels in Hct116 PDCs without GA, without MG132, or with both GA and MG132 treatment. **b** Vimentin expression levels in LoVo PDCs without GA, without MG132, and with both GA and MG132 treatments. **I** (**a**) Total vimentin expression levels in Hct116 PDCs before and after mutation at the SUMO sites (K373/K439/K445, K373/K439, K439/K445, and K373/K445). **b** Total vimentin expression levels in LoVo PDCs before and after mutation at the SUMO site (K373/K439/K445, K373/K439, K439/K445, K373 /K445). *SUMO* small ubiquitin-like modification, *PDCs* polyploid giant cancer cells with daughter cells, *GA* ginkgolic acid

These results suggest that SUMOylation promotes nuclear translocation of vimentin. To confirm the relationship between SUMOylation and vimentin expression, siRNA was used to suppress the expression of SUMO1. After SUMO1 (Figs. 2F, G and 3A, B) and SUMO2/3 (Fig. 3C–F) knockdown in PDCs, the total, cytoplasmic, and nuclear vimentin expression levels decreased, and the differences between SUMO1 knockout groups compared to the NC groups were statistically significant (Additional file 1: Fig. S1F, G, S2A, B). In addition, the same results were obtained using the SUMOylation inhibitor GA (Fig. 3G). Vimentin expression levels were significantly decreased in PDCs after GA treatment compared to PDCs without GA treatment (Additional file 4: Fig. S2C), which interrupts the formation of the phase-activating enzyme E1-SUMO thioester complex. Low vimentin expression in GA-treated PDCs was rescued by MG132, a proteasome inhibitor that protects ubiquitinated proteins from proteasome-mediated degradation. The use of MG132 alone resulted in no significant elevation of vimentin expression compared with that in the blank group, which suggests that vimentin SUMOylation in PDCs reduces the ubiquitinated degradation of vimentin (Fig. 3H, a and b ). The expression level of vimentin in PDCs with GA, both GA, and MG132 treatment was less than that in PDCs, and the differences were statistically significant (Additional file 4: Fig. S2D).

**Fig. 4:**
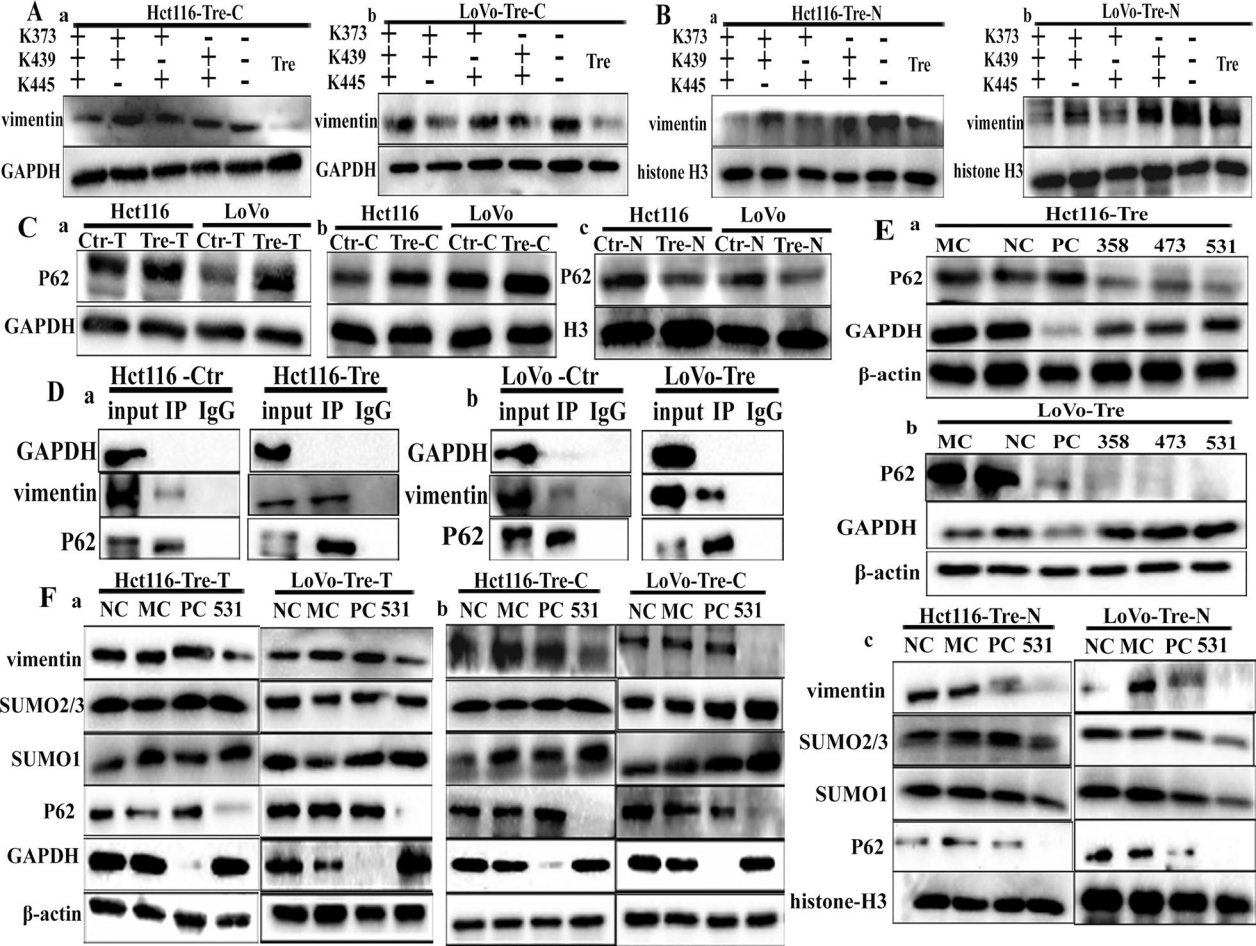
**A** (**a**) Cytoplasmic vimentin expression levels in Hct116 PDCs before and after mutation of the SUMO site (K373/K439/K445, K373/K439, K439/K445, and K373/K445). **b** Cytoplasmic vimentin expression levels in LoVo PDCs before and after mutation of the SUMO site (K373/K439/K445, K373/K439, K439/K445, and K373/K445). **B** (**a**) Nuclear vimentin expression levels in Hct116 PDCs before and after mutations at the SUMO site (K373/K439/K445, K373/K439, K439/K445, and K373/K445); **b** nuclear vimentin expression levels in LoVo PDCs before and after mutations at the SUMO site (K373/K439/K445, K373/K439, K439/K445, and K373/K445). **C** Western blot analysis of P62 protein levels. **a** Total protein level of P62, **b** cytoplasmic level of P62, and **c** nuclear level of P62. **D** (**a**) Results of vimentin coimmunoprecipitation in Hct116 control cells and Hct116 PDCs (anti-vimentin antibody was used for immunoprecipitation). **b** Results of vimentin coimmunoprecipitation in LoVo control cells and LoVo PDCs (anti-vimentin antibody was used for immunoprecipitation). **E** (**a**) Total P62 expression levels in Hct116 PDCs after siRNA-P62 (358, 473, 531) transfection. **b** Total P62 expression levels in LoVo PDCs after siRNA-P62 (358, 473, 531) transfection. **F** (**a**) Total vimentin, SUMO1, and SUMO2/3 expression levels after siRNA-P62. **b** Cytoplasmic vimentin, SUMO1, and SUMO2/3 expression levels after siRNA-P62. **c** Nuclear vimentin, SUMO1, and SUMO2/3 expression levels after siRNA-P62. *SUMO* small ubiquitin-like modification, *PDCs* polyploid giant cancer cells with daughter cells

Quantitative proteomic experiments have shown that the SUMOylation sites of vimentin occur at K439 and K445, both of which are located in the tail domain and are highly conserved across species [21]. In addition, analysis using SUMO 2.0 prediction software showed that vimentin was most likely to undergo SUMOylation at K375. To clarify the molecular mechanism by which SUMOylated vimentin enters the nucleus of PDCs, different combinations of vimentin lysine mutation sites (K375, K439, and K445R), including single (Additional file 4: Figs. S2E, F, S3A, B), double, and triple mutants (VIM^mt^) and overexpressed wild-type vimentin (VIM^wt^), were evaluated. Subsequently, we compared the functional effects of PDCs transfected with an empty overexpression vector as a negative control. The WB blotting showed that vimentin was abundant in the total protein sample (Fig. 3I, a and b) and was slightly reduced in the cytoplasmic fraction (Fig. 4A, a and b). Compared to VIM^wt^, VIM^mt^ nuclear expression was significantly reduced, with the most pronounced changes observed in the triple mutant (Fig. 4B, a and b) (Additional file 4: Fig. S3C, D). To determine the effect of inhibiting vimentin nuclear translocation by evaluating the impact of vimentin SUMOylation on cell invasion and migration, Transwell assays were used to compare the invasion and migration abilities of PDCs after VIM^mt^ and VIM^wt^ transfection. The VIM^mt^ results revealed a significant inhibition of cell invasion and migration (Additional file 4: Fig. S3E, F), and the average cell number per field in the VIM^mt^ sequence groups decreased compared with that in the VIM^wt^ groups (Additional file 4: Fig. S3G) and the differences were statistically significant.

### Up-regulated P62 promotes vimentin nuclear localization by regulating SUMO1 and SUMO2/3 expression

**Fig. 5:**
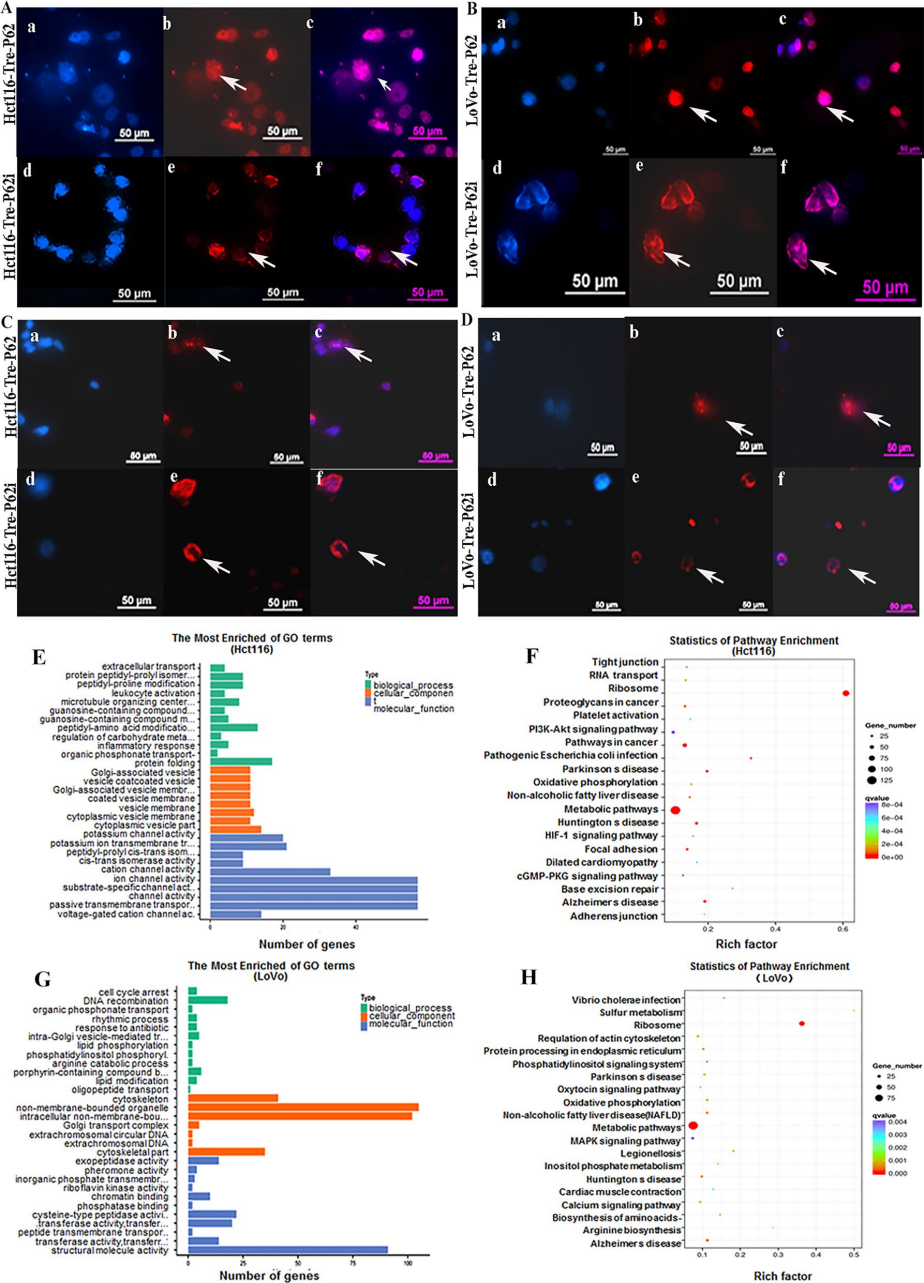
**A** SUMO2/3 expression in Hct116 PDCs before and after siRNA-P62 transfection. **B** SUMO2/3 expression levels in LoVo PDCs before and after siRNA-P62 transfection. **C** SUMO1 expression in Hct116 PDCs before and after siRNA-P62 transfection. **D** SUMO1 expression levels in LoVo PDCs before and after siRNA-P62 transfection. **E** GO functional enrichment of targets associated with vimentin in the Hct116 PDC ChIP-seq peak evaluation of biological processes. **F** KEGG pathway clustering analysis of targets associated with vimentin in Hct116 PDC from ChIP-seq peaks. **G** GO functional enrichment of targets associated with vimentin in analysis of LoVo PDC ChIP-seq peaks for biological processes. **H** KEGG pathway clustering analysis of targets associated with vimentin in the LoVo PDC ChIP-seq peaks.* SUMO* small ubiquitin-like modification, *PDCs* polyploid giant cancer cells with daughter cells, *GO* Gene Ontology, *ChIP* chromatin immunoprecipitation

P62 plays an essential role as a scaffolding protein in protein transport. To assess the role of P62 in nuclear localization of vimentin in PDCs, the expression and subcellular localization of P62 were detected in Hct116 and LoVo control cells and PDCs. Although total and cytoplasmic P62 were overexpressed in PDCs (Fig. 4C, a and b), P62 nuclear expression was significantly suppressed (Fig. 4C, c and Additional file 4: Fig. S4A). The results of the co-immunoprecipitation experiments suggested that vimentin interacted with P62 (Fig. 4D, a and b). Since vimentin levels are elevated and P62 levels are decreased in the nuclei of PDCs, we evaluated the mechanism by which P62 regulates the nuclear localization of vimentin. After P62 expression was knocked down (Fig. 4E, a and b) in PDCs, total, cytoplasmic, and nuclear vimentin expression levels decreased (Fig. 4F). In addition, the cytoplasmic expression levels of SUMO1 and SUMO2/3 were slightly increased in Hct116 and LoVo PDCs after P62 knockdown (Fig. 4F, b and Additional file 4: Fig. S4D, 4E), and SUMO1 and SUMO2/3 expression was also reduced in the nucleus (Fig. 4F, c) (*P* < 0.01, Additional file 4: Fig. S4F, S5A, B). Immunofluorescence staining was performed to investigate the effect of P62 on the subcellular localization of SUMO1 and SUMO2/3. When P62 was knocked down, SUMO1 (Fig. 5A, B) and SUMO2/3 (Fig. 5C, D) expression in PDCs decreased in the nucleus and increased in the cytoplasm. This finding suggests that P62 is essential for maintaining the nuclear expression of SUMO1 and SUMO2/3. Furthermore, these results suggested that upregulated P62 acts as a scaffold that indirectly regulates the nuclear translocation of vimentin by controlling the subcellular localization of SUMO1 and SUMO2/3.

### Vimentin acts as a nuclear transcription factor and promotes cell migration and invasion via CDC42-cathepsin B and D

P62-dependent vimentin nuclear translocation is a consequence of SUMO pathway activation, and nuclear vimentin is associated with PDC migration and invasion. A ChIP assay was performed using an antibody against vimentin, followed by sequencing to assess the potential targets of vimentin. The results showed 3670 significant ChIP-seq peaks in Hct116-derived PDCs (Additional file 2: Table S9) and 1649 in LoVo-derived PDCs (Additional file 3: Table S10). In addition, GO analysis implicated vimentin in cell migration (Fig. 5E, G), and KEGG pathway enrichment analysis implicated vimentin in tumor-invasive metastasis (Fig. 5F, H). Among these genes, *CDC42* and *ANXA10* are upregulated in various human cancer cell lines and their expression correlates with tumor stage, lymph node metastasis, and patient survival. ChIP sequence analysis also confirmed that vimentin binds to the *CDC42* promoter.

**Fig. 6:**
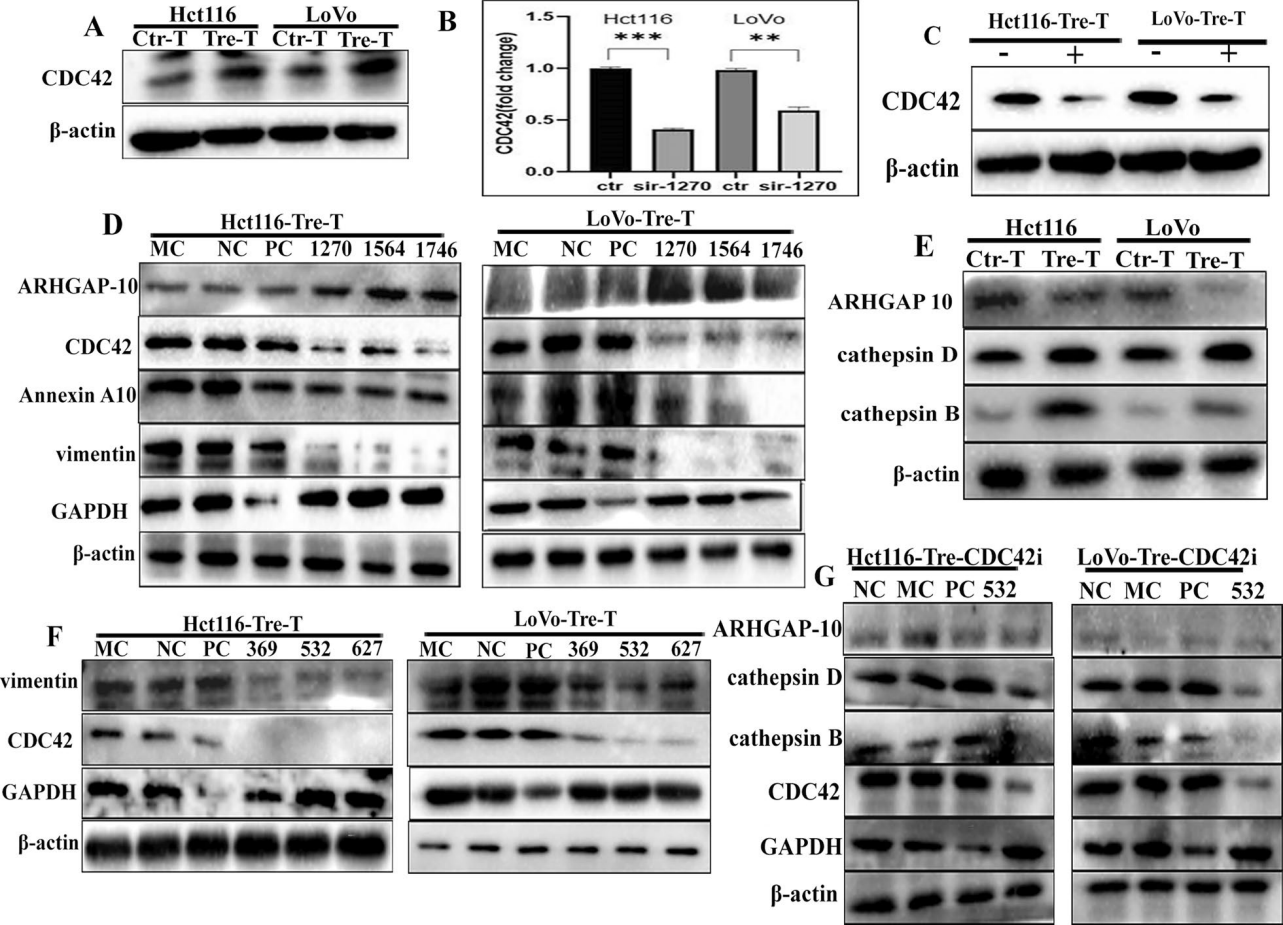
**A** CDC42 expression in Hct116 and LoVo cells before and after treatment. **B** CDC42 expression in Hct116 and LoVo PDCs after siRNA-vimentin (1270) transfection. **C** CDC42 expression in Hct116 and LoVo PDCs after GA. **D** ARHGAP10, CDC42, and Annexin A10 expression levels in Hct116 and LoVo PDCs after siRNA-vimentin (1270, 1564, 1746) transfection. **E** ARHGAP10 and cathepsin B and D expression in Hct116 and LoVo cells before and after treatment. **F** Vimentin expression levels in Hct116 and LoVo PDCs after siRNA-CDC42 (369, 532, 627) transfection. **G** Expression of cathepsin B, cathepsin D, and ARHGAP10 in Hct116 and LoVo PDCs after siRNA-CDC42 (532) transfection. *PDCs* polyploid giant cancer cells with daughter cells, *ARHGAP10* rho-guanine nucleotide exchange factor 18

We also examined the expression of CDC42, and the results revealed that CDC42 expression was elevated in PDCs compared to control cells (Fig. 6A and Additional file 4: Fig. S5C, a). qPCR was performed simultaneously and the *CDC42* RNA level decreased after vimentin knockdown, indicating that vimentin may act as a transcription factor that promote *CDC42* gene expression (Fig. 6B). The expression of CDC42 was also reduced in PDCs after GA treatment (Additional file 4: Fig. S5C, b), further suggesting that SUMOylated vimentin entered the nucleus and regulated *CDC42* gene expression (Fig. 6C).

To further investigate the relationship between vimentin and CDC42, siRNA was used to inhibit vimentin expression in PDCs. Subsequent WB results showed that CDC42 and Annexin A10 protein levels decreased after vimentin knockdown, whereas Rho-guanine nucleotide exchange factor 18 (ARHGAP10) levels were elevated (Fig. 6D). Another unpublished study reported that CDC42 combined with cathepsin B and D promotes the migration and invasion of CoCl_2_-induced PDCs. In this study, we also found that the expression of cathepsin B and D increased and ARHGAP10 expression decreased in PDCs after treatment compared to control cells (Fig. 6E). To further investigate the effect of CDC42 on cell migration, we examined the expression levels of cathepsin B, cathepsin D, and ARHGAP10 in PDCs after CDC42 knockdown (Fig. 6F, G). The WB blotting revealed that the expression of cathepsin B and D decreased after CDC42 knockdown (Additional file 4: Fig. S5D, E). Furthermore, Luo et al. reported that ARHGAP10 interacts with CDC42 and that ARHGAP10 overexpression inhibits CDC42 activity [28]. In the present study, the expression of ARHGAP10 remained unchanged in PDCs before and after CDC42 knockdown (Fig. 8D). Subsequent transwell assays showed that CDC42 knockdown significantly decreased cell invasion and metastasis (Additional file 4: Fig. S5F, G), and the average cell number per field in the CDC42 knockout groups was significantly lower than that in the NC groups (Additional file 4: Fig. S5H). Based on these results, we hypothesized that nuclear vimentin acts as a transcription factor of CDC42 and promotes cell migration via the vimentin-ARHGAP10-CDC42-cathepsin B and D signaling pathways.

### Expression of vimentin and its related proteins in xenografts

**Fig. 7:**
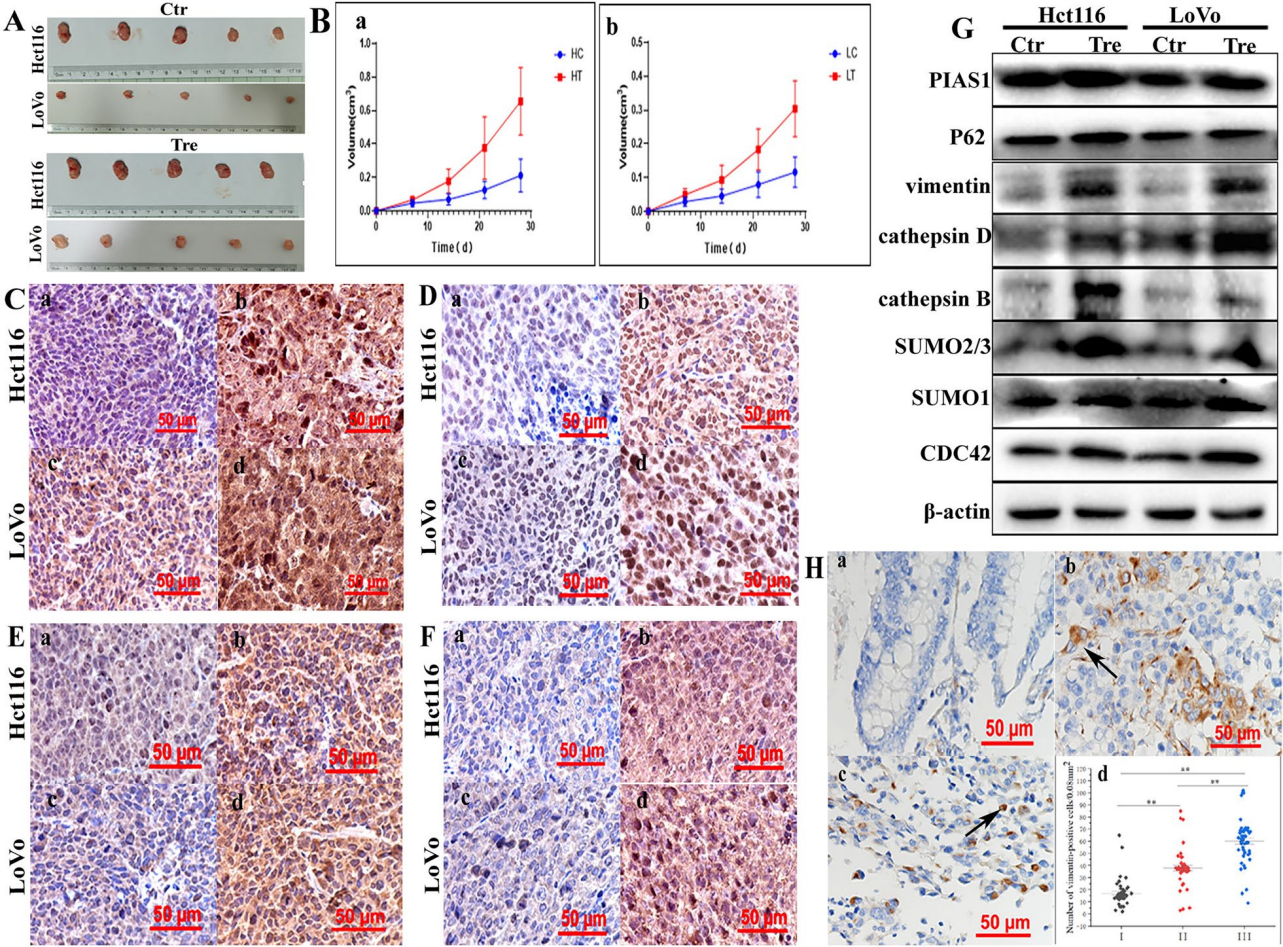
**A** Images showing tumor tissues inoculated with Hct116 and LoVo cells before and after treatment. **B** Growth curves of xenograft-inoculated **a** Hct116 and **b** LoVo cells with and without treatment. IHC staining of **C** vimentin, **D** SUMO2/3, **E** P62, and **F** CDC42 in tumor tissues inoculated with Hct116 and LoVo cells before and after treatment (×200). **a** Hct116 control cells and **B** Hct116 PDCs. **c** LoVo control cells, **d** LoVo PDCs. **G** Vimentin-related total protein levels (including PIAS1, P62, vimentin, cathepsin B, cathepsin D, SUMO1, SUMO2/3, and CDC42) in tumor tissues inoculated with Hct116 and LoVo cells treated with and without treatment shown in the WB assay. **H** Vimentin expression in **a** well-differentiated, **b** moderately differentiated, and **c** poorly differentiated colorectal cancer tissues. **d** Number of vimentin-positive cells/0.08 mm^2^ in (I) ell-differentiated, (II) moderately differentiated, and (III) poorly differentiated colorectal cancer tissues. *SUMO* small ubiquitin-like modification, *PDCs* polyploid giant cancer cells with daughter cells, *IHC* immunohistochemical

Xenografts were obtained by the subcutaneous injection of Hct116 and LoVo control cells and PDCs. At 28 days after inoculation, all mice were euthanized and tumor tissues were used for IHC and WB analyses. Analysis of tumor growth curves showed that the mean volume of xenograft tumors inoculated with PDCs was significantly higher than that of tumors inoculated with control cells (Fig. 7A, B), confirming enhanced tumorigenesis by PDCs. Morphological features were observed by hematoxylin and eosin staining. Compared to xenografts from the control group, cells with more meganuclei and multinuclei were observed in tumors from the PDC groups (Additional file 4: Fig. S5I). The IHC results showed that the PDC group was positive for vimentin and showed higher nuclear positivity (Fig. 7C). In addition, SUMO2/3 (Fig. 7D), P62 (Fig. 7E), and CDC42 (Fig. 7F) showed a greater degree of staining in the PDC group than in the control group, and the staining indices of SUMO2/3, P62, and CDC42 were significantly higher in the PDCs xenografts than in the control xenografts (*P* < 0.01, Additional file 4: Fig. S6A, a, b, c and d). Furthermore, the WB analysis showed that the expression levels of vimentin (Additional file 4: Fig. S6B, a), PIAS1 (Additional file 4: Fig. S6B, b), P62 (Additional file 4: Fig. S6B, c), cathepsin B and D (Additional file 4: Fig. S6B, d and E), SUMO2/3 (Additional file 4: Fig. S6B, f), SUMO1 (Additional file 4: Fig. S6B, g), and CDC42 (Additional file 4: Fig. S6B, h) were lower in the xenograft tumor tissues of Hct116 and LoVo control cells than in the PDCs (Fig. 7G). These results suggested that PDCs have a stronger tumorigenic capacity, which may be related to the high expression of vimentin in the nucleus.

**Fig. 8:**
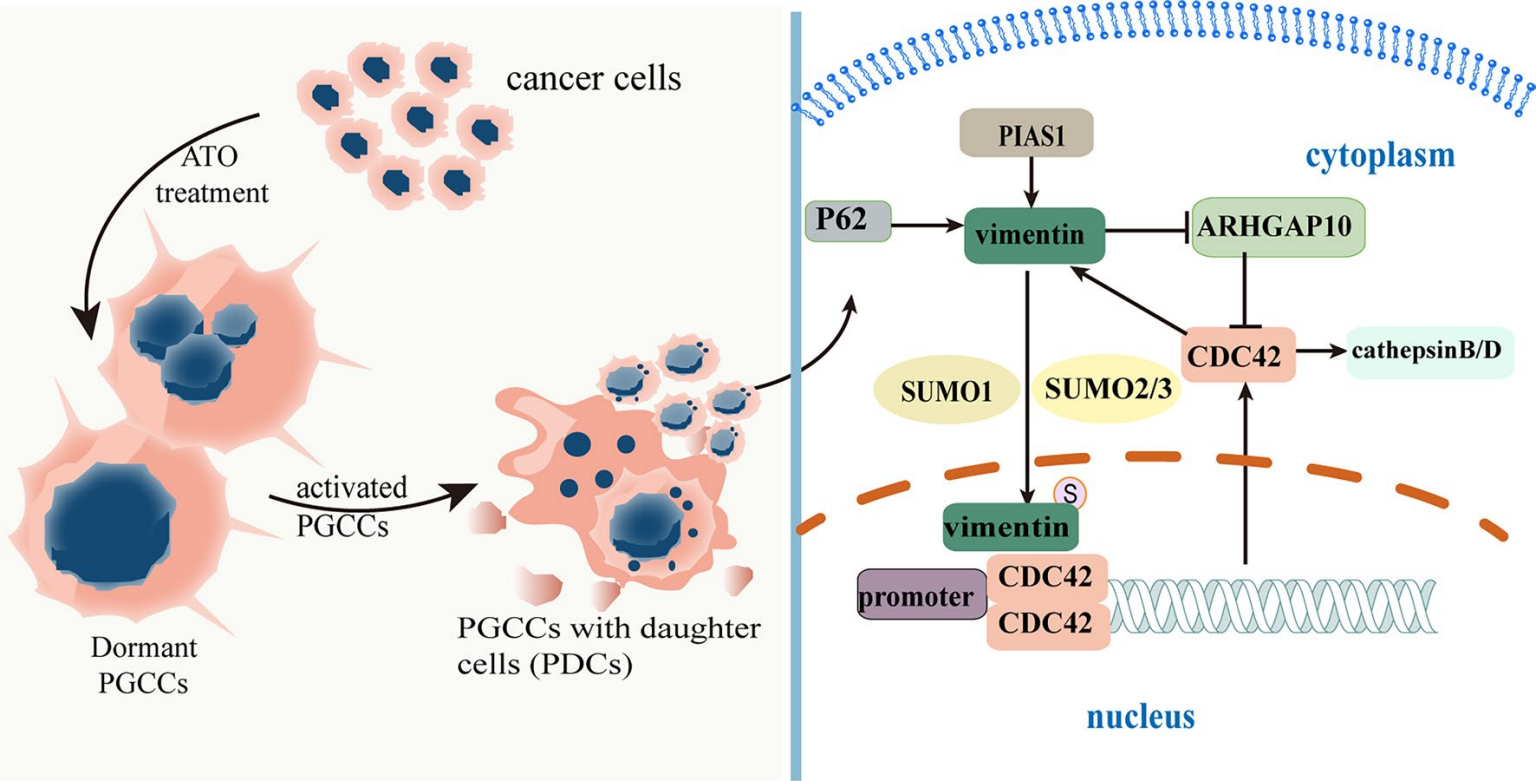
The mechanism by which nuclear-located vimentin regulates PDC invasion, proliferation, and migration. Vimentin is modified by SUMOylation in PDCs after treatment and cannot be degraded by the proteasome, resulting in increased vimentin expression in PDCs. Upregulated P62 regulates the subcellular localization of vimentin by controlling the movement of SUMO1 and SUMO2/3. Nuclear vimentin acts as a transcription factor and promotes cell migration via the CDC42-cathepsin B- and D-vimentin pathways. *SUMO* small ubiquitin-like modification, *PDCs* polyploid giant cancer cells with daughter cells

### Vimentin nuclear localization is a valuable biomarker for CRC differentiation

Because cancer cells may promote tumor progression, recurrence, and metastasis by producing PDCs and other forms of heterogeneous tumor cells after exposure to chemical reagents, hypoxia, and radiotherapy, we confirmed the presence of PDCs, which were defined as cells with irregular meganuclei or multiple nuclei, in human CRC H&E-stained sections. PDCs present in CRC tissues express fewer epithelial markers and acquire a mesenchymal phenotype with increased vimentin expression. Furthermore, the nuclear expression of vimentin was significantly higher in poorly differentiated specimens (Fig. 7H, c) than in moderately differentiated (Fig. 7H, b) and highly differentiated specimens (Fig. 7F, a). In addition, the number of PDCs was significantly higher in poorly differentiated specimens than in moderately and highly differentiated specimens (Fig. 7F, d) (Table 1). These findings suggest that more PDCs and higher vimentin nuclear translocation levels are correlated with the degree of CRC differentiation and worse clinical prognosis.

**Table 1:** The number of PGCCs in different group of human CRCs

	Group	n	Number of PGCCs/0.08 mm^2^	Value of statistics	*P*
Well-differentiated CRCs	Group I	55	6.76 ± 1.73	χ^2^ =119.04	0.000*
Moderately differentiated CRCs	Group II	50	9.66 ± 2.20		
Poorly differentiated CRCs	Group III	50	18.66 ± 1.70		

*P < 0.05: statistically significant. (P: difference among the three groups; P1 (difference between groups I and II) = 0.002; P2 (difference between groups II and III) = 0.000; P3 (difference between groups I and III) = 0.000

## Discussion

CSCs can develop resistance to cancer treatments and can evade immune surveillance [29]. Most recurrent and disseminated metastatic events are caused by the reactivation of CSCs [30, 31]. PGCCs have unique morphological characteristics and can be considered CSCs under various unfavorable conditions [6]. As a specific CSCs type, PGCCs contribute to the heterogeneity of solid tumors [32]. Hypoxia-mimetic CoCl_2_, chemical reagents, radiotherapy, and Chinese herbal medicine can induce PGCCs formation by regulating the expression of both cell cycle-and cell fusion-related proteins [7, 8, 33–36]. Under unfavorable microenvironment, PGCCs switch from “mitotic arrest” to dormant state, which enable PGCCs to survival in the harsh microenvironments. When these unfavorable microenvironments disappear, PGCCs can produce daughter cells via asymmetric divisions, such as budding and bursting [37].

In this study, PGCCs were induced by ATO. After a brief cell cycle arrest, PGCCs began to produce daughter cells (PDCs). Moreover, PDCs exhibit greater proliferation, invasion, and metastatic capacities [8, 38]. In addition, PDCs can repopulate and form macroscopic colonies in vitro while generating tumor volume faster than normal diploid cells when inoculated into mice [39]. The mouse xenografts used in this study also showed that PDCs had a greater capacity for tumor formation. Furthermore, results from 155 patients with CRC showed that higher levels of PDCs and vimentin nuclear translocation correlated with the degree of CRC differentiation.

Vimentin, a cytoskeletal protein, is widely present in various cells, tissues, and organs and is necessary for normal cell survival and growth [40, 41]. The expression and subcellular localization of vimentin are essential for cell movement, which ultimately leads to cancer recurrence and metastasis via SUMOylation of vimentin [42, 43]. In the present study, PDCs from CRC cell lines with elevated vimentin expression showed changes in subcellular localization due to vimentin SUMOylation. Our transwell assay results revealed that cells transfected with VIM^mt^ had significantly reduced invasion and metastatic abilities compared to those transfected with VIM^wt^, which was consistent with the SUMO knockdown and GA results. These findings are consistent with recent studies showing that vimentin SUMOylation promotes tumor migration in breast cancer cells [32]. Interestingly, our findings suggest that the transcription of certain key proteins is involved in the entry of vimentin into the nucleus. To elucidate the correlation among PGCCs, the generation of more invasive and metastatic daughter cells, and vimentin nuclear translocation, the results of the ChIP-seq analysis and subsequent experiments suggest that vimentin acts as a transcription factor that promotes the migration of PDCs through the vimentin-ARHGAP10-CDC42-cathepsin B and D signaling pathway. Notably, cathepsin D can activate the precursor of cathepsin B, which further activates cathepsin D and transports cathepsins B and D between cells through the actin skeleton and microtubules, thereby degrading the extracellular matrix and promoting cancer cell migration and invasion [38]. In this study, we confirmed that the expression of cathepsin B and D was regulated by CDC42 and was higher in PDCs than in control cells. ARHGAP10 is a member of the RhoGAP protein family and its overexpression of ARHGAP10 inhibits the activity of CDC42 by converting the GTP-bound form into the GDP-bound form [28]. In our study, the expression of ARHGAP10 was lower in PDCs than in control cells; however, ARHGAP10 expression remained unchanged in PDCs before and after CDC42 knockdown.

The signaling junction P62 is a multistructural domain protein that activates multiple transcription factors, including NF-κB, and regulates cellular autophagy, placing P62 at a critical point in controlling cell death and survival [44]. P62 is required for the formation of perinuclear and nuclear aggregates that contain SUMO 2/3 chains [45]. P62 and SUMO2/3 are proteins implicated in the ubiquitin-proteasome system, and their levels are stimulated in a stress-dependent manner. The expression of P62, SUMO1, SUMO2/3 and PIAS1 was upregulated, which appears to be in agreement with the increase in SUMO conjugation activity observed in response to various stimuli, such as ATO treatment [46]. Our results suggest that vimentin SUMOylation causes its nuclear translocation in a P62-dependent manner. The elevation of P62 levels in PGCCs to generate daughter cells implies that targeting P62 may be a strategy to inhibit PGCCs.

## Conclusions

Our study elucidated the roles of vimentin SUMOylation and P62 upregulation in PDC invasion and migration. The detailed molecular mechanism by which nuclear localization of vimentin regulates PDC invasion and migration is shown in Fig. 8. Targeting nuclear vimentin may be a promising therapeutic strategy against metastatic cancers. This strategy may be used to develop effective therapies that target vimentin nuclear translocation and P62 to prevent or eradicate PGCCs and generate daughter cells with enhanced invasive metastatic properties. In conclusion, our data provide new insights into the clinical applications of therapies targeting PGCCs in daughter cells.

### Supplementary Information

**Additional file 1: Table S1.** Detail information of antibodies used in the paper. **Table S2.** SUMO1-siRNA interfering sequences. **Table S3.** SUMO2-siRNA interfering sequences. **Table S4.** SUMO3-siRNA interfering sequences. **Table S5.** P62-siRNA interfering sequences. **Table S6.** Vimentin-siRNA interfering sequences. **Table S7.** CDC42-siRNA interfering sequences. **Table S8.** CDC42 primer sequences.

**Additional file 2: Table S9.** Hct116_narrow_peaks_ChIPseeker_annotation.

**Additional file 3: Table S10.** LoVo_narrow_peaks_ChIPseeker_annotation.

**Additional file 4: Figure S1.** **A** Column diagram showing the comparison of the abilities of Wound-healing (P < 0.05, P < 0.01). **B** the colony formation efficiency (a) in Hct116 and (b) LoVo cells with and without ATO treatment (P < 0.05, P < 0.01). Gray value analysis of western blots.All data represent the means ± standard errors of means of at least three independent experiments. (a) Total protein, (b) plasma protein, and (c) nuclear protein. **C** PIAS1, **D** SUMO1, **E** SUMO2/3. All data represent the means ± standard errors of means of at least three independent experiments. (a) plasma protein, and (b) nuclear protein. **F** Vimentin expression in Hct116 PDCs before and after siRNA-SUMO1(727).**G** Vimentin expression in LoVo PDCs before and after siRNA-SUMO1(727). *P* values are calculated using the one-way analysis of variance. *P < 0.05; **P < 0.01; ***P < 0.001. **Figure S2.** Gray value analysis of western blots. All data represent the means ± standard errors of means of at least three independent experiments. (a) Total protein, (b) plasma protein, and (c) nuclear protein. **A** Vimentin expression in Hct116 PDCs before and after siRNA-SUMO2/3(498 814). **B** Vimentin expression in LoVo PDCs before and after siRNA-SUMO2/3(498 814). **C** Vimentin expression levels in Hct116 PDCs and LoVo PDCs after GA. **D** Vimentin expression levels in Hct116 PDCs without GA, without MG132, and both GA and MG132 treatments (P < 0.05, P < 0.01). **E** Vimentin expression levels in LoVo PDCs without GA, without MG132, and both GA and MG132 treatments (P < 0.05, P < 0.01). **F** Vimentin expression in Hct116 PDCs before and after mutations at the SUMO site (K373, K439, K445). (a) Total vimentin expression level, (b) cytoplasmic vimentin expression level, and (c) nuclear vimentin expression level. **G** Vimentin expression in LoVo PDCs before and after mutations at the SUMO site (K373, K439, K445). (a) Total vimentin expression level, (b) cytoplasmic vimentin expression level, and (c) nuclear vimentin expression level. *P* values are calculated using the one-way analysis of variance. *P < 0.05; **P < 0.01; ***P < 0.001. **Figure S3.** Gray value analysis of western blots. All data represent the means ± standard errors of means of at least three independent experiments. (a) Total protein, (b) plasma protein, and (c) nuclear protein. **A** Vimentin expression in Hct116 PDCs before and after mutations at the SUMO site (K373, K439, K445). **B** Vimentin expression in LoVo PDCs before and after mutations at the SUMO site (K373, K439, K445). **C** Vimentin expression levels in Hct116 PDCs before and after mutations at the SUMO site (K373/K439/K445, K373/K439, K439/K445, K373/K445). **D** Vimentin expression levels in LoVo PDCs before and after mutations at the SUMO site (K373/K439/K445, K373/K439, K439/K445, K373 /K445). **E** The migration abilities of Hct116 and LoVo PDCs before and after mutation for 24 h (100×). **F** The invasion abilities of Hct116 and LoVo PDCs before and after mutation for 24 h (100×). **G** (a) Migration efficiency in Hct116 and LoVo PDCs before and after mutation. (b) The invasion abilities efficiency in Hct116 and LoVo PDCs before and after mutation (P < 0.05, P < 0.01). *P* values are calculated using the one-way analysis of variance. *P < 0.05; **P < 0.01; ***P < 0.001. **Figure S4.** Gray value analysis of western blots. All data represent the means ± standard errors of means of at least three independent experiments. **A** (a) Total protein levels of P62, (b) cytoplasmic levels of P62, and (c) nuclear levels of P62. **B** Total protein expression levels of vimentin, SUMO1,and SUMO2/3 in Hct116 PDCs before and after siRNA-P62 transfection. **C** Total protein expression levels of vimentin, SUMO1,and SUMO2/3 in LoVo PDCs before and after siRNA-P62 transfection. **D** Plasma protein expression levels of vimentin, SUMO1,and SUMO2/3 in Hct116 PDCs before and after siRNA-P62 transfection. **E** Plasma protein expression levels of vimentin, SUMO1,and SUMO2/3 in LoVo PDCs before and after siRNA-P62 transfection.**F** Nuclear protein expression levels of vimentin in Hct116 PDCs before and after siRNA-P62 transfection. *P* values are calculated using the one-way analysis of variance. *P < 0.05; **P < 0.01; ***P < 0.001. **Figure S5.** Gray value analysis of western blots. All data represent the means ± standard errors of means of at least three independent experiments.**A** Nuclear protein expression levels of SUMO1and SUMO2/3 in Hct116 PDCs before and after siRNA-P62 transfection. **B** Nuclear protein expression levels of vimentin, SUMO1,and SUMO2/3 in LoVo PDCs before and after siRNA-P62 transfection. **C** (a) CDC42 expression levels in Hct116 and LoVo cells before and after ATO. (b) CDC42 expression levels in Hct116 and LoVo PDCs before and after GA. **D** Expression levels of cathepsin D and cathepsin B in Hct116 PDCs before and after siRNA-CDC(532). **E** Expression levels of cathepsin D and cathepsin B in LoVo PDCs before and after siRNA-CDC(532). **F** Cell invasion assay in Hct116 and LoVo PDCs after siRNA-CDC42 (532) transfection (200×). **G** Cell migration assay in Hct116 and LoVo PDCs after siRNA-CDC42 (532) transfection (200×). **H** (a) The invasion abilities efficiency in Hct116 and LoVo PDCs before and after mutation. (b) Migration efficiency in Hct116 and LoVo PDCs before and after mutation. (P < 0.05, P < 0.01). **I** H&E staining of tumor tissues inoculated with Hct116 and LoVo cells before and after ATO treatment (200×). PDCs, polyploid giant cancer cells with daughter cells; ATO, arsenic trioxide; H&E, hematoxylin and eosin. *P* values are calculated using the one-way analysis of variance. *P < 0.05; **P < 0.01; ***P < 0.001. **Figure S6.** Staning index analysis of immunochemistry.All data represent the means ± standard errors of means of at least three independent experiments. **A** (a) vimentin, (b) SUMO2/3, (c) P62, and (d) CDC42. Gray value analysis of western blots. All data represent the means ± standard errors of means of at least three independent experiments. **B** (a) PIAS1, (b) P62, (c) vimentin, (d) cathepsin D. (e) cathepsin B, (f) CDC42, (g) SUMO2/3, and (h) SUMO1. *P* values are calculated using the one-way analysis of variance. *P < 0.05; **P < 0.01; ***P < 0.001.

## Data Availability

The authors declare that all data supporting the findings of this study are available within the article and its Additional files or by contacting the corresponding author upon reasonable request.

## Abbreviations

PGCCs: Polyploid giant cancer cells
PDCs: PGCCs with daughter cells
CSCs: Cancer stem cells
ATO: Arsenic trioxide
ICC: Immunocytochemistry
IHC: Immunohistochemistry
CRC: Colorectal cancer
SUMOylation: Small ubiquitin-like modifications
SUMO: Small ubiquitin-like modifier proteins
PBS: Phosphate buffered saline
GA: Ginkgolic acid
WB: Western blot
GO: Gene ontology
