# Supplementary material for: Molecular mechanism of vimentin nuclear localization associated with the migration and invasion of daughter cells derived from polyploid giant cancer cells
Source: J Transl Med. 2023 Oct 13;21:719. doi: 10.1186/s12967-023-04585-7 (PMC10576317; doi:10.1186/s12967-023-04585-7)
Supplement: Supplementary file 1 — Additional file 1: Table S1. Detail information of antibodies used in the paper. Table S2. SUMO1-siRNA interfering sequences. Table S3. SUMO2-siRNA interfering sequences. Table S4. SUMO3-siRNA interfering sequences. Table S5. P62-siRNA interfering sequences. Table S6. Vimentin-siRNA interfering sequences. Table S7. CDC42-siRNA interfering sequences. Table S8. CDC42 primer sequences. [file 12967_2023_4585_MOESM1_ESM.docx]

Table S1. Detail information of antibodies used in the paper.

| protein | Species specificity | Company | WB | ICC/IHC | | IF |
| --- | --- | --- | --- | --- | --- | --- |
| Vimentin | Mouse monoclonal | Proteintech | 1:1000 | 1:1000 |  | |
| Vimentin | Rabbit Polyclonal | Thermo | 1:1000 |  |  | |
| ANXA10 | Rabbit polyclonal | Affinity | 1:500 |  | - | |
| P62 | Mouse monoclonal | Proteintech | 1:1000 | 1:1000 | - | |
| SUMO1 | Mouse monoclonal | Proteintech | 1:1000 |  | 1:500 | |
| PIAS1 | Rabbit monoclonal | Proteintech | 1:1000 | 1:1000 | - | |
| CDC42 | Rabbit monoclonal | Proteintech | 1:1000 | 1:1000 | - | |
| ARHGEP10 | Rabbit polyclonal | Affinity | 1:500 | - | - | |
| GAPDH | Rabbit monoclonal | CST | 1:3000 | - | - | |
| β-actin | Mouse monoclonal | Proteintech | 1:1000 | - | - | |
| SUMO2/3 | Mouse monoclonal | Proteintech | 1:1000 | 1:1000 | 1:500 | |
| CathepsinB | Rabbit polyclonal | Affinity | 1:2000 |  |  | |
| CathepsinD | Rabbit polyclonal | Boster | 1:1000 | 1:1000 |  | |

Table S2. SUMO1-siRNA interfering sequences.

| **Names** | **Sense (5ʹ-3ʹ)** | **Antisense (5ʹ-3ʹ)** |
| --- | --- | --- |
| **SUMO1-307** | GACAGGGUGUUCCAAUGAATT | UUCAUUGGAACACCCUGUCTT |
| **SUMO1-358** | GAGAAUUGCUGAUAAUCAUTT | AUGAUUAUCAGCAAUUCUCTT |
| **SUMO1-727** | GGCUUGUGGUGAUAAAUAATT | UUAUUUAUCACCACAAGCCTT |
| **SUMO1-GAPDH** | UGACCUCAACUACAUGGUUTT | AACCAUGUAGUUGAGGUCATT |
| **SUMO1-NC** | UUCUCCGAACGUGUCACGUTT | ACGUGACACGUUCGGAGAATT |

Table S3. SUMO2-siRNA interfering sequences.

| **Names** | **Sense (5ʹ-3ʹ)** | **Antisense (5ʹ-3ʹ)** |
| --- | --- | --- |
| **SUMO2-275** | GCAUACACCACUUAGUAAATT | UUUACUAAGUGGUGUAUGCTT |
| **SUMO2-315** | CGACAGGGAUUGUCAAUGATT | UCAUUGACAAUCCCUGUCGTT |
| **SUMO2-498** | GACCAAGAUUACAUUCUCATT | UGAGAAUGUAAUCUUGGUCTT |
| **SUMO2-GAPDH** | UGACCUCAACUACAUGGUUTT | AACCAUGUAGUUGAGGUCATT |
| **SUMO2-NC** | UUCUCCGAACGUGUCACGUTT | ACGUGACACGUUCGGAGAATT |

Table S4. SUMO3-siRNA interfering sequences.

| **Names** | **Sense (5ʹ-3ʹ)** | **Antisense (5ʹ-3ʹ)** |
| --- | --- | --- |
| **SUMO3-471** | CAAUGAAACUGACACUCCATT | UGGAGUGUCAGUUUCAUUGTT |
| **SUMO3-744** | CUGCAGGGAUGAAUCUGUATT | UACAGAUUCAUCCCUGCAGTT |
| **SUMO3-814** | GCAAGAUAUUGUGGGUACUTT | AGUACCCACAAUAUCUUGCTT |
| **SUMO3-GAPDH** | UGACCUCAACUACAUGGUUTT | AACCAUGUAGUUGAGGUCATT |
| **SUMO3-NC** | UUCUCCGAACGUGUCACGUTT | ACGUGACACGUUCGGAGAATT |

Table S5. P62-siRNA interfering sequences.

| **Names** | **Sense (5ʹ-3ʹ)** | **Antisense (5ʹ-3ʹ)** |
| --- | --- | --- |
| **P62-358** | CCUACGUGAAGGAUGACAUTT | AUGUCAUCCUUCACGUAGGTT |
| **P62-473** | GAUCUGCGAUGGCUGCAAUTT | AUUGCAGCCAUCGCAGAUCTT |
| **P62-531** | CCAGACUACGACUUGUGUATT | UACACAAGUCGUAGUCUGGTT |
| **P62-GAPDH** | UGACCUCAACUACAUGGUUTT | AACCAUGUAGUUGAGGUCATT |
| **P62-NC** | UUCUCCGAACGUGUCACGUTT | ACGUGACACGUUCGGAGAATT |

Table S6. Vimentin--siRNA interfering sequences.

| **Names** | **Sense (5ʹ-3ʹ)** | **Antisense (5ʹ-3ʹ)** |
| --- | --- | --- |
| **vimentin-1270** | GCAGAAGAAUGGUACAAAUTT | AUUUGUACCAUUCUUCUGCTT |
| **vimentin-1564** | GACCUGCUCAAUGUUAAGATT | UCUUAACAUUGAGCAGGUCTT |
| **vimentin-1746** | GACGGUUGAAACUAGAGAUTT | AUCUCUAGUUUCAACCGUCTT |
| **vimentin-GAPDH** | UGACCUCAACUACAUGGUUTT | AACCAUGUAGUUGAGGUCATT |
| **vimentin-NC** | UUCUCCGAACGUGUCACGUTT | ACGUGACACGUUCGGAGAATT |

Table S7. CDC42-siRNA interfering sequences.

| **Names** | **Sense (5ʹ-3ʹ)** | **Antisense (5ʹ-3ʹ)** |
| --- | --- | --- |
| **CDC42-369** | CCGCUGAGUUAUCCACAAATT | UUUGUGGAUAACUCAGCGGTT |
| **CDC42-532** | CCUCUACUAUUGAGAAACUTT | UACAGAUUCAUCCCUGCAGTT |
| **CDC42-627** | GUGGAGUGUUCUGCACUUATT | UAAGUGCAGAACACUCCACTT |
| **CDC42-GAPDH** | UGACCUCAACUACAUGGUUTT | AACCAUGUAGUUGAGGUCATT |
| **CDC42-NC** | UUCUCCGAACGUGUCACGUTT | ACGUGACACGUUCGGAGAATT |

Table S8. *CDC42* primer sequences

| **Names** | **Sense (5ʹ-3ʹ)** | **Antisense (5ʹ-3ʹ)** |
| --- | --- | --- |
| ***CDC42*** | CCATCGGAATATGTACCGACG | CTCAGCGGTCGAAATCTGTCA |
