# Supplementary material for: Molecular mechanism of vimentin nuclear localization associated with the migration and invasion of daughter cells derived from polyploid giant cancer cells
Source: J Transl Med. 2023 Oct 13;21:719. doi: 10.1186/s12967-023-04585-7 (PMC10576317; doi:10.1186/s12967-023-04585-7)
Supplement: Supplementary file 4 — Additional file 4: Figure S1. A Column diagram showing the comparison of the abilities of Wound-healing (P < 0.05, P < 0.01). B the colony formation efficiency (a) in Hct116 and (b) LoVo cells with and without ATO treatment (P < 0.05, P < 0.01). Gray value analysis of western blots.All data represent the means ± standard errors of means of at least three independent experiments. (a) Total protein, (b) plasma protein, and (c) nuclear protein. C PIAS1, D SUMO1, E SUMO2/3. All data represent the means ± standard errors of means of at least three independent experiments. (a) plasma protein, and (b) nuclear protein. F Vimentin expression in Hct116 PDCs before and after siRNA-SUMO1(727).G Vimentin expression in LoVo PDCs before and after siRNA-SUMO1(727). P values are calculated using the one-way analysis of variance. *P < 0.05; **P < 0.01; ***P < 0.001. Figure S2. Gray value analysis of western blots. All data represent the means ± standard errors of means of at least three independent experiments. (a) Total protein, (b) plasma protein, and (c) nuclear protein. A Vimentin expression in Hct116 PDCs before and after siRNA-SUMO2/3(498 814). B Vimentin expression in LoVo PDCs before and after siRNA-SUMO2/3(498 814). C Vimentin expression levels in Hct116 PDCs and LoVo PDCs after GA. D Vimentin expression levels in Hct116 PDCs without GA, without MG132, and both GA and MG132 treatments (P < 0.05, P < 0.01). E Vimentin expression levels in LoVo PDCs without GA, without MG132, and both GA and MG132 treatments (P < 0.05, P < 0.01). F Vimentin expression in Hct116 PDCs before and after mutations at the SUMO site (K373, K439, K445). (a) Total vimentin expression level, (b) cytoplasmic vimentin expression level, and (c) nuclear vimentin expression level. G Vimentin expression in LoVo PDCs before and after mutations at the SUMO site (K373, K439, K445). (a) Total vimentin expression level, (b) cytoplasmic vimentin expression level, and (c) nuclear vimentin expression level. P values [file 12967_2023_4585_MOESM4_ESM.docx]

**Additional figures and figure legends**

**
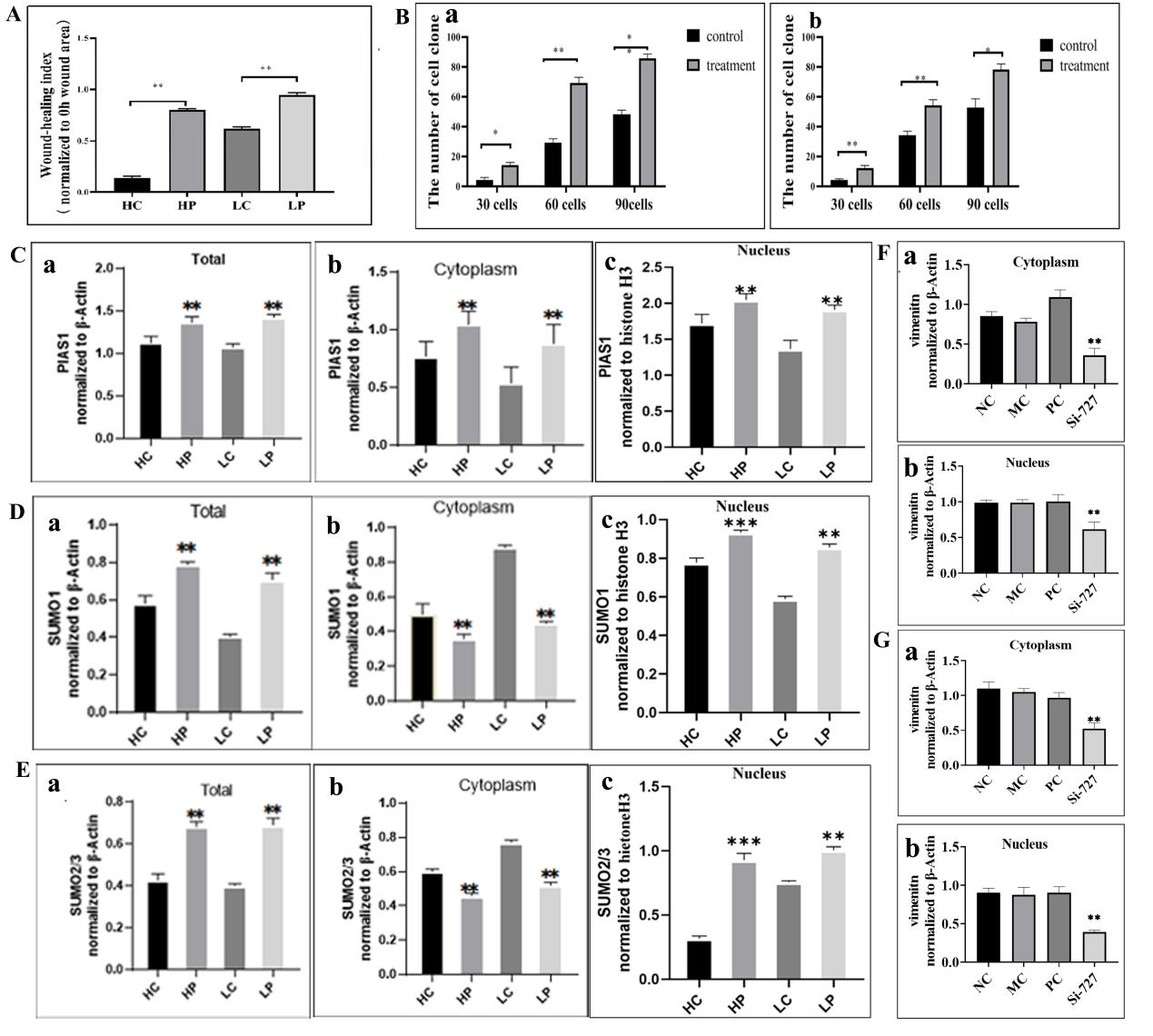
**

**Figure S1. A.** Column diagram showing the comparison of the abilities of Wound-healing ( P < 0.05,P < 0.01). **B.** the colony formation efficiency (a) in Hct116 and (b) LoVo cells with and without ATO treatment ( P < 0.05,P < 0.01). Gray value analysis of western blots. All data represent the means ± standard errors of means of at least three independent experiments. (a) Total protein, (b) plasma protein, and (c) nuclear protein. **C.** PIAS1, **D.** SUMO1, **E.** SUMO2/3. All data represent the means ± standard errors of means of at least three independent experiments. (a) plasma protein, and (b) nuclear protein. **F.** Vimentin expression in Hct116 PDCs before and after siRNA-SUMO1(727). **G.** Vimentin expression in LoVo PDCs before and after siRNA-SUMO1(727). P values are calculated using the one-way analysis of variance. *, P < 0.05; **, P < 0.01; ***, P < 0.001.

SUMO, small ubiquitin-like modification; PDCs, polyploid giant cancer cells with daughter cells; ATO, arsenic trioxide; HC, Hct116 control cells; HP, Hct116 PDCs after ATO treatment; LC, LoVo control cells; LP, LoVo PDCs after ATO treatment.


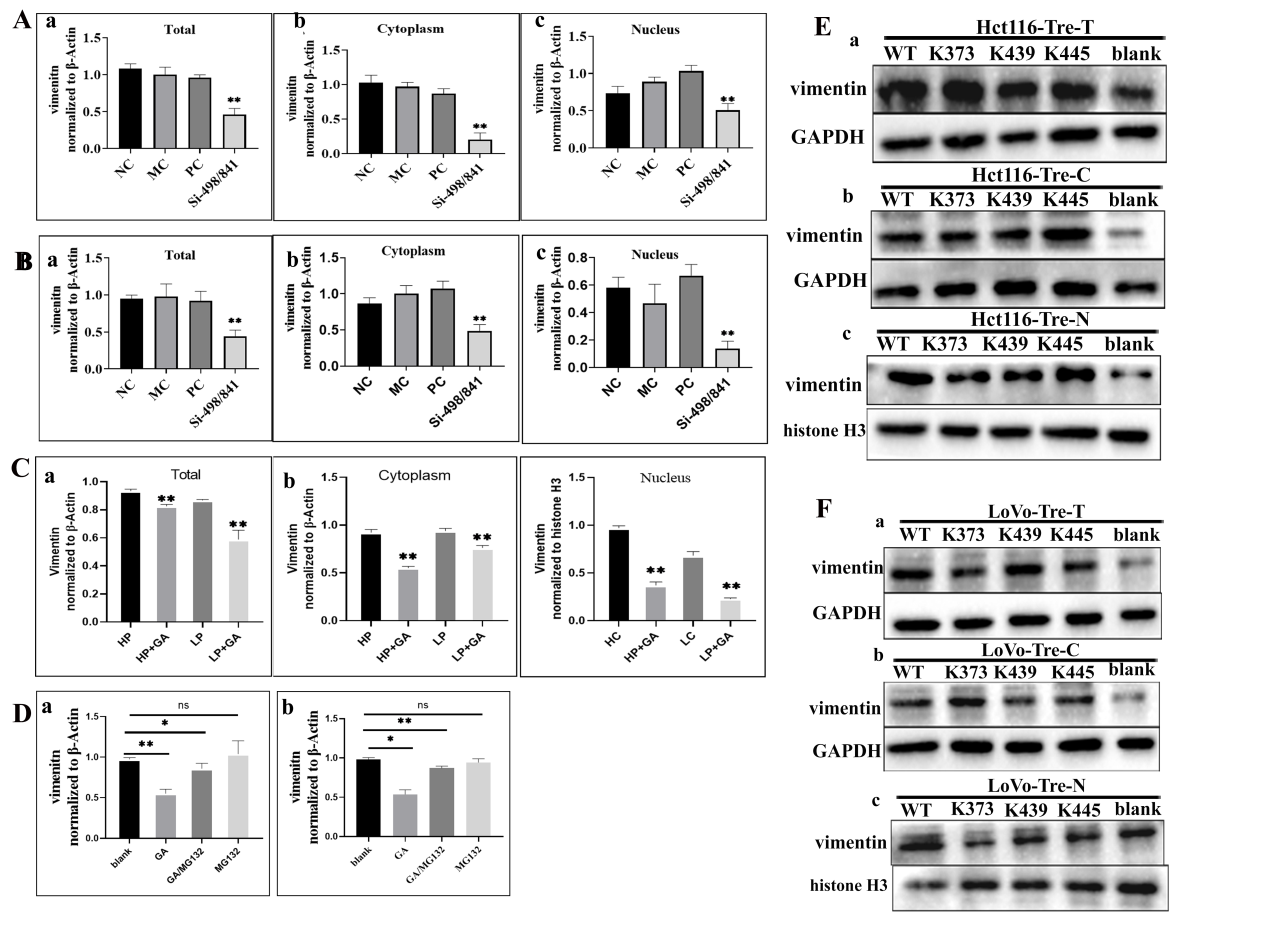


**Figure S2.** **Gray value analysis of western blots**. All data represent the means ± standard errors of means of at least three independent experiments. (a) Total protein, (b) plasma protein, and (c) nuclear protein. **A.** Vimentin expression in Hct116 PDCs before and after siRNA-SUMO2/3(498 814). **B.** Vimentin expression in LoVo PDCs before and after siRNA-SUMO2/3(498 814). **C.** Vimentin expression levels in Hct116 PDCs and LoVo PDCs after GA. **D.** Vimentin expression levels in Hct116 PDCs without GA, without MG132, and both GA and MG132 treatments ( P < 0.05,P < 0.01). **E.** Vimentin expression levels in LoVo PDCs without GA, without MG132, and both GA and MG132 treatments ( P < 0.05,P < 0.01). **F**. Vimentin expression in Hct116 PDCs before and after mutations at the SUMO site (K373, K439, K445). (a) Total vimentin expression level, (b) cytoplasmic vimentin expression level, and (c) nuclear vimentin expression level. **G.** Vimentin expression in LoVo PDCs before and after mutations at the SUMO site (K373, K439, K445). (a) Total vimentin expression level, (b) cytoplasmic vimentin expression level, and (c) nuclear vimentin expression level. P values are calculated using the one-way analysis of variance. *, P < 0.05; **, P < 0.01; ***, P < 0.001.

SUMO, small ubiquitin-like modification; PDCs, polyploid giant cancer cells with daughter cells; ATO, arsenic trioxide; HC, Hct116 control cells; HP, Hct116 PDCs after ATO treatment; LC, LoVo control cells; LP, LoVo PDCs after ATO treatment; WT, wild type; GA, Ginkgolic acid.


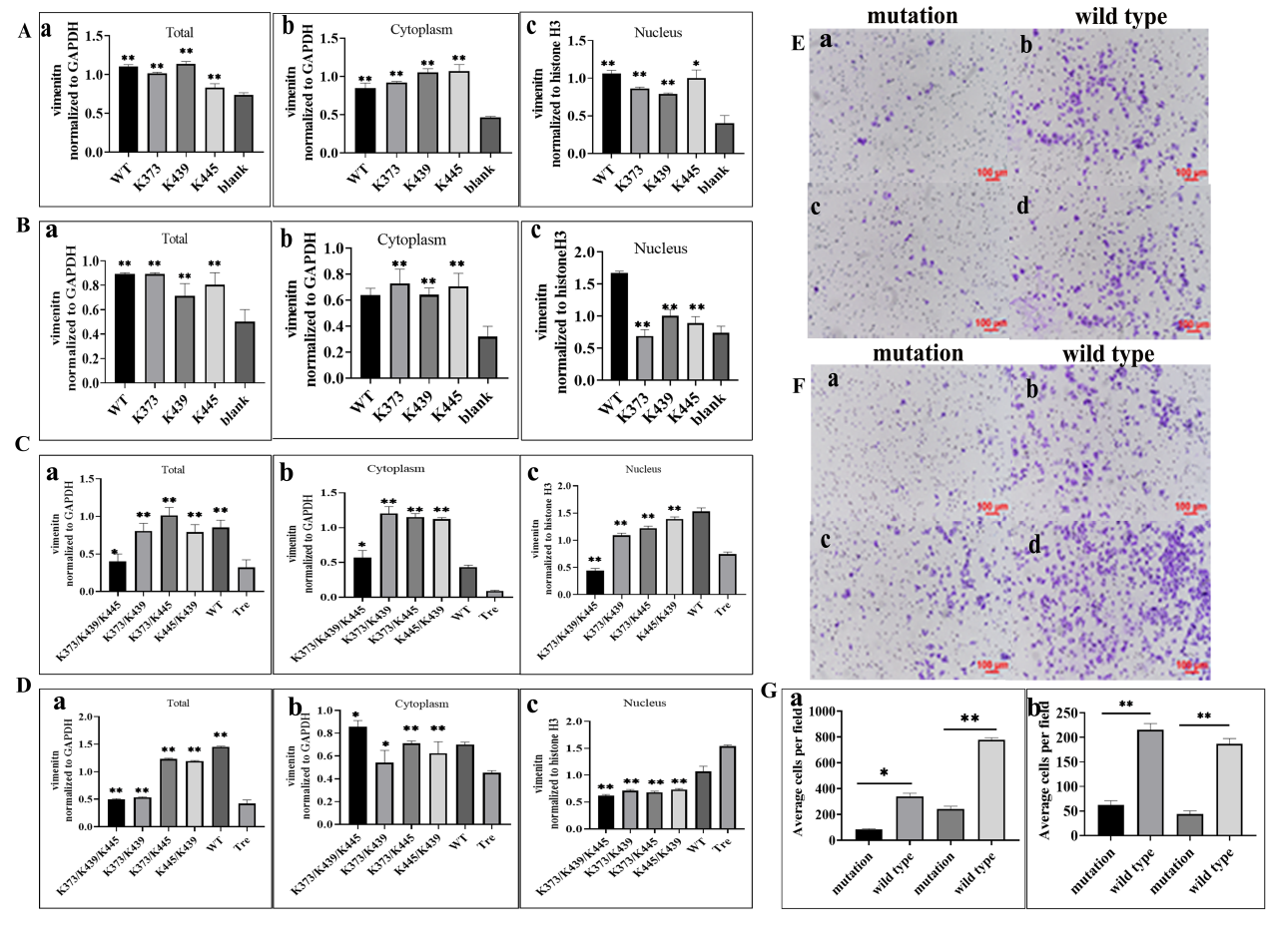


**Figure S3. Gray value analysis of western blots.** All data represent the means ± standard errors of means of at least three independent experiments. (a) Total protein, (b) plasma protein, and (c) nuclear protein. **A**. Vimentin expression in Hct116 PDCs before and after mutations at the SUMO site (K373, K439, K445). **B.** Vimentin expression in LoVo PDCs before and after mutations at the SUMO site (K373, K439, K445). **C.**Vimentin expression levels in Hct116 PDCs before and after mutations at the SUMO site (K373/K439/K445, K373/K439, K439/K445, K373/K445). **D.** Vimentin expression levels in LoVo PDCs before and after mutations at the SUMO site (K373/K439/K445, K373/K439, K439/K445, K373 /K445). **E.** The migration abilities of Hct116 and LoVo PDCs before and after mutation for 24 h (100X). **F.** The invasion abilities of Hct116 and LoVo PDCs before and after mutation for 24 h (100×). **G.** (a) Migration efficiency in Hct116 and LoVo PDCs before and after mutation. (b) The invasion abilities efficiency in Hct116 and LoVo PDCs before and after mutation ( P < 0.05,P < 0.01). P values are calculated using the one-way analysis of variance. *, P < 0.05; **, P < 0.01; ***, P < 0.001.


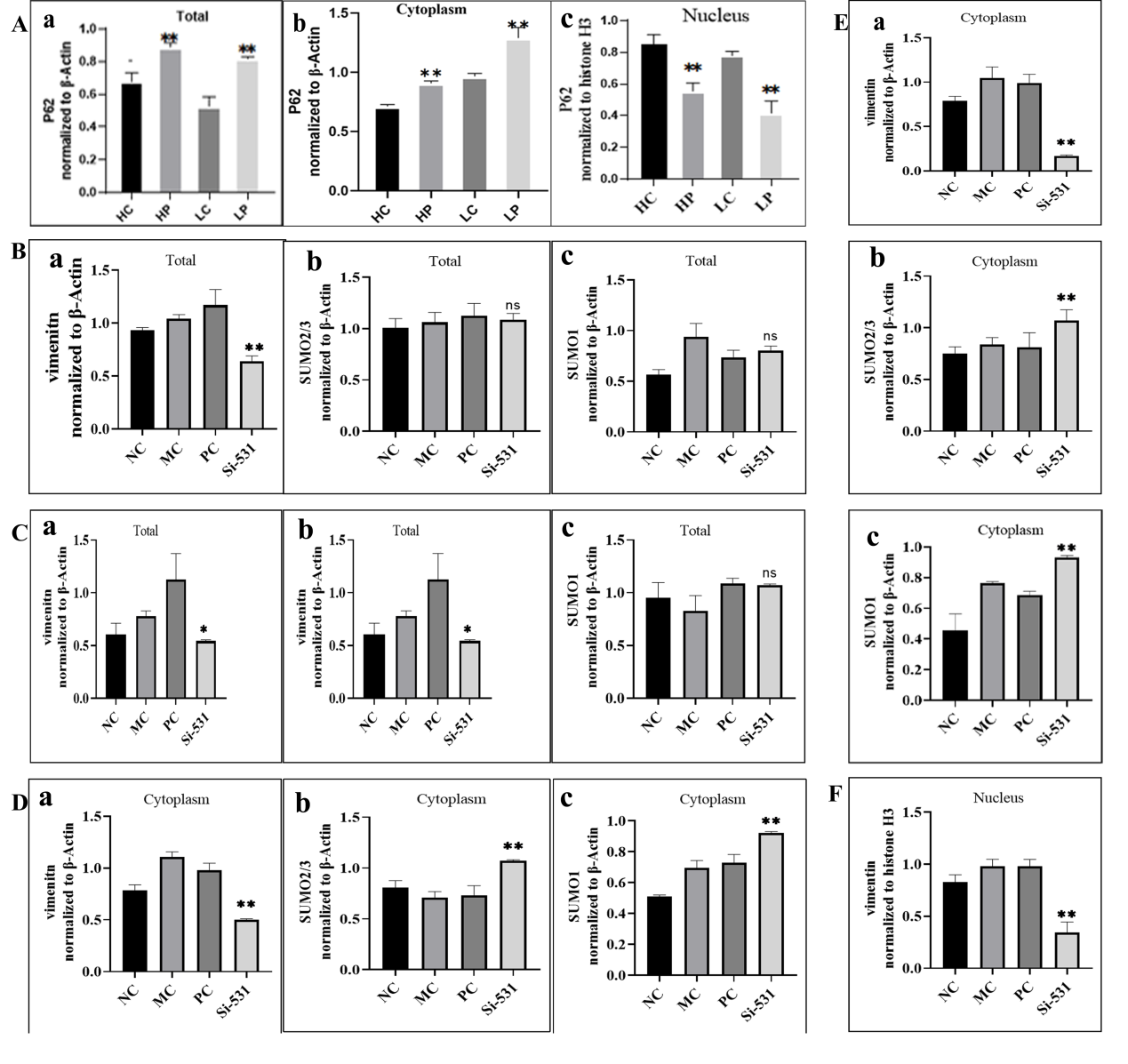


**Figure S4. Gray value analysis of western blots.** All data represent the means ± standard errors of means of at least three independent experiments. **A.**(a) Total protein levels of P62, (b) cytoplasmic levels of P62, and (c) nuclear levels of P62. **B.** Total protein expression levels of vimentin, SUMO1,and SUMO2/3 in Hct116 PDCs before and after siRNA-P62 transfection. **C.** Total protein expression levels of vimentin, SUMO1,and SUMO2/3 in LoVo PDCs before and after siRNA-P62 transfection. **D.** Plasma protein expression levels of vimentin, SUMO1,and SUMO2/3 in Hct116 PDCs before and after siRNA-P62 transfection. **E.** Plasma protein expression levels of vimentin, SUMO1,and SUMO2/3 in LoVo PDCs before and after siRNA-P62 transfection. **F.** Nuclear protein expression levels of vimentin in Hct116 PDCs before and after siRNA-P62 transfection. P values are calculated using the one-way analysis of variance. *, P < 0.05; **, P < 0.01; ***, P < 0.001.

SUMO, small ubiquitin-like modification; PDCs, polyploid giant cancer cells with daughter cells; ATO, arsenic trioxide; HC, Hct116 control cells; HP, Hct116 PDCs after ATO treatment; LC, LoVo control cells; LP, LoVo PDCs after ATO treatment.


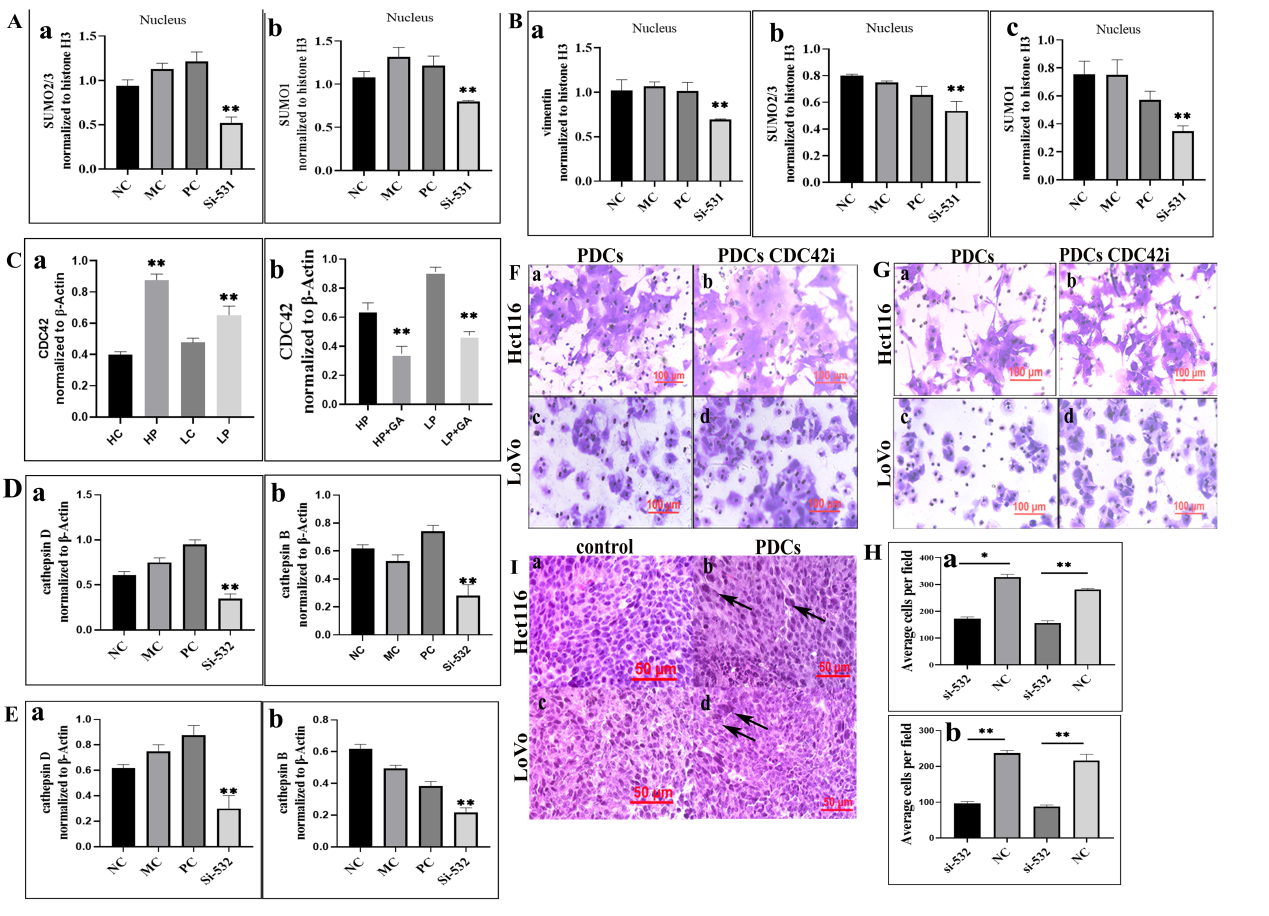


**Figure S5. Gray value analysis of western blots.** All data represent the means ± standard errors of means of at least three independent experiments. **A.** Nuclear protein expression levels of SUMO1and SUMO2/3 in Hct116 PDCs before and after siRNA-P62 transfection. **B.** Nuclear protein expression levels of vimentin, SUMO1,and SUMO2/3 in LoVo PDCs before and after siRNA-P62 transfection. **C.** (a) CDC42 expression levels in Hct116 and LoVo cells before and after ATO. (b) CDC42 expression levels in Hct116 and LoVo PDCs before and after GA. **D.** Expression levels of cathepsin D and cathepsin B in Hct116 PDCs before and after siRNA-CDC(532). **E.** Expression levels of cathepsin D and cathepsin B in LoVo PDCs before and after siRNA-CDC(532). **F.** Cell invasion assay in Hct116 and LoVo PDCs after siRNA-CDC42 (532) transfection (200X). **G.** Cell migration assay in Hct116 and LoVo PDCs after siRNA-CDC42 (532) transfection (200X). **H.** (a) The invasion abilities efficiency in Hct116 and LoVo PDCs before and after mutation. (b) Migration efficiency in Hct116 and LoVo PDCs before and after mutation. ( P < 0.05, P < 0.01). **I.** H&E staining of tumor tissues inoculated with Hct116 and LoVo cells before and after ATO treatment (200X). PDCs, polyploid giant cancer cells with daughter cells; ATO, arsenic trioxide; H&E, hematoxylin and eosin. P values are calculated using the one-way analysis of variance. *, P < 0.05; **, P < 0.01; ***, P < 0.001.

SUMO, small ubiquitin-like modification; PDCs, polyploid giant cancer cells with daughter cells; ATO, arsenic trioxide;


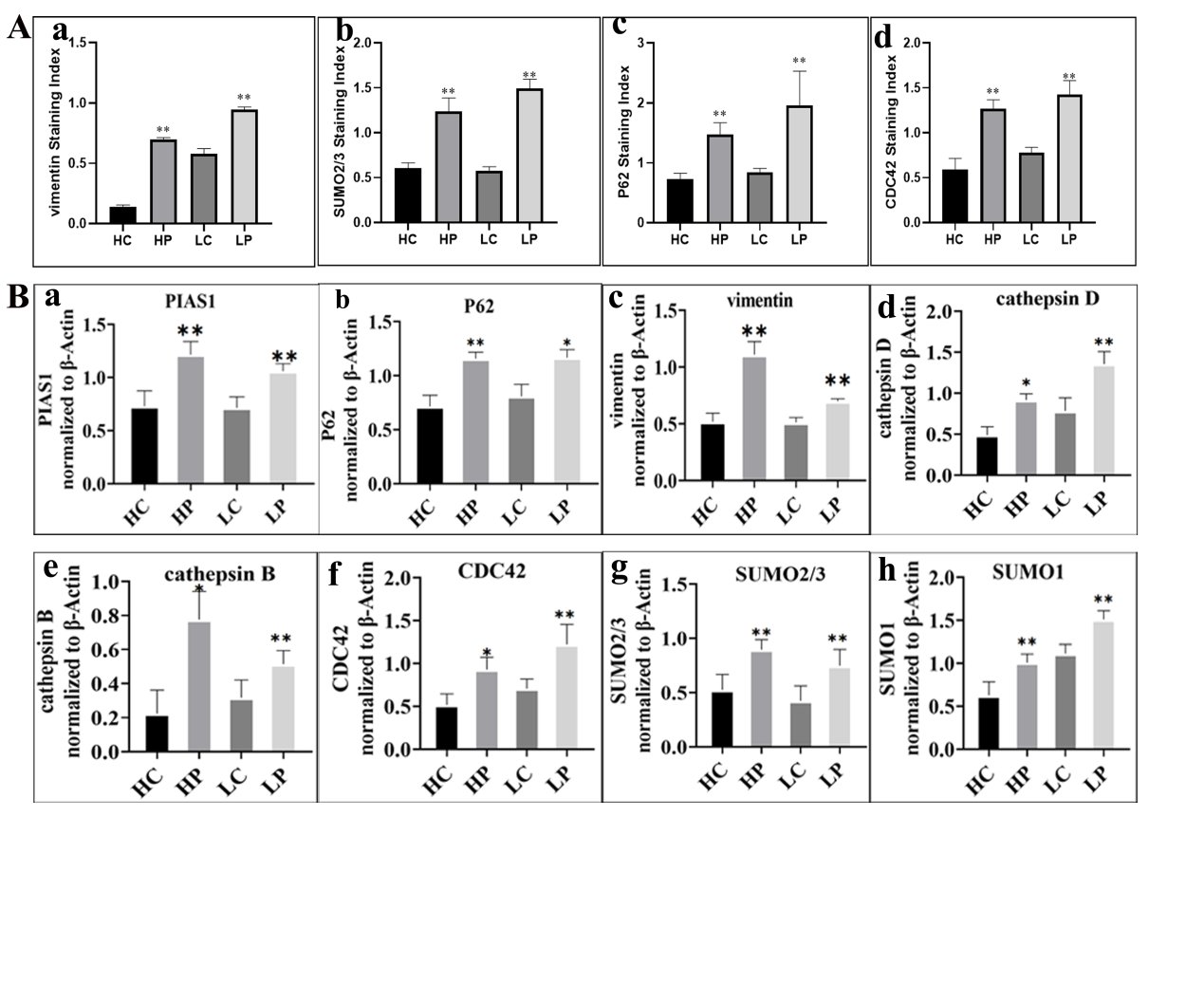


**Figure S6.** Staning index analysis of immunochemistry. All data represent the means ± standard errors of means of at least three independent experiments. **A.** (a) vimentin, (b) SUMO2/3, (c) P62, and (d) CDC42. Gray value analysis of western blots. All data represent the means ± standard errors of means of at least three independent experiments.**B.** (a) PIAS1, (b) P62, (c) vimentin, (d) cathepsin D. (e) cathepsin B, (f) CDC42, (g) SUMO2/3, and (h) SUMO1. P values are calculated using the one-way analysis of variance. *, P < 0.05; **, P < 0.01; ***, P < 0.001.

SUMO, small ubiquitin-like modification; HC, Hct116 control cells; HP, Hct116 PDCs after ATO treatment; LC, LoVo control cells; LP, LoVo PDCs after ATO treatment.
